# Engineering the Biosynthesis of Late-Stage Vinblastine Precursors Precondylocarpine Acetate, Catharanthine, Tabersonine in *Nicotiana benthamiana*

**DOI:** 10.1021/acssynbio.2c00434

**Published:** 2022-12-14

**Authors:** Dagny Grzech, Benke Hong, Lorenzo Caputi, Prashant D. Sonawane, Sarah E. O’Connor

**Affiliations:** † Department of Natural Product Biosynthesis, Max Planck Institute for Chemical Ecology, 07745 Jena, Germany

## Abstract

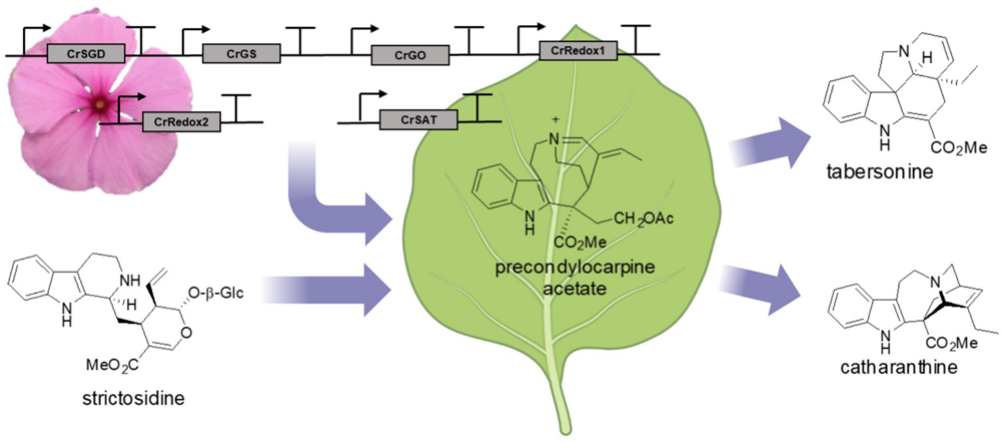

Vinblastine is a chemotherapy agent produced by the plant *Catharanthus roseus* in small quantities. Currently, vinblastine is sourced by isolation or semisynthesis. *Nicotiana benthamiana* is a plant heterologous host that can be used for reconstitution of biosynthetic pathways as an alternative natural product sourcing strategy. Recently, the biosynthesis of the late-stage vinblastine precursors precondylocarpine acetate, catharanthine, and tabersonine have been fully elucidated. However, the large number of enzymes involved in the pathway and the unstable nature of intermediates make the reconstitution of late-stage vinblastine precursor biosynthesis challenging. We used the *N. benthamiana* chassis and a state-of-art modular vector assembly to optimize the six biosynthetic steps leading to production of precondylocarpine acetate from the central intermediate strictosidine (∼2.7 mg per 1 g frozen tissue). After selecting the optimal regulatory element combination, we constructed four transcriptional unit assemblies and tested their efficiency. Finally, we successfully reconstituted the biosynthetic steps leading to production of catharanthine and tabersonine.

## Introduction

**Figure 1:**
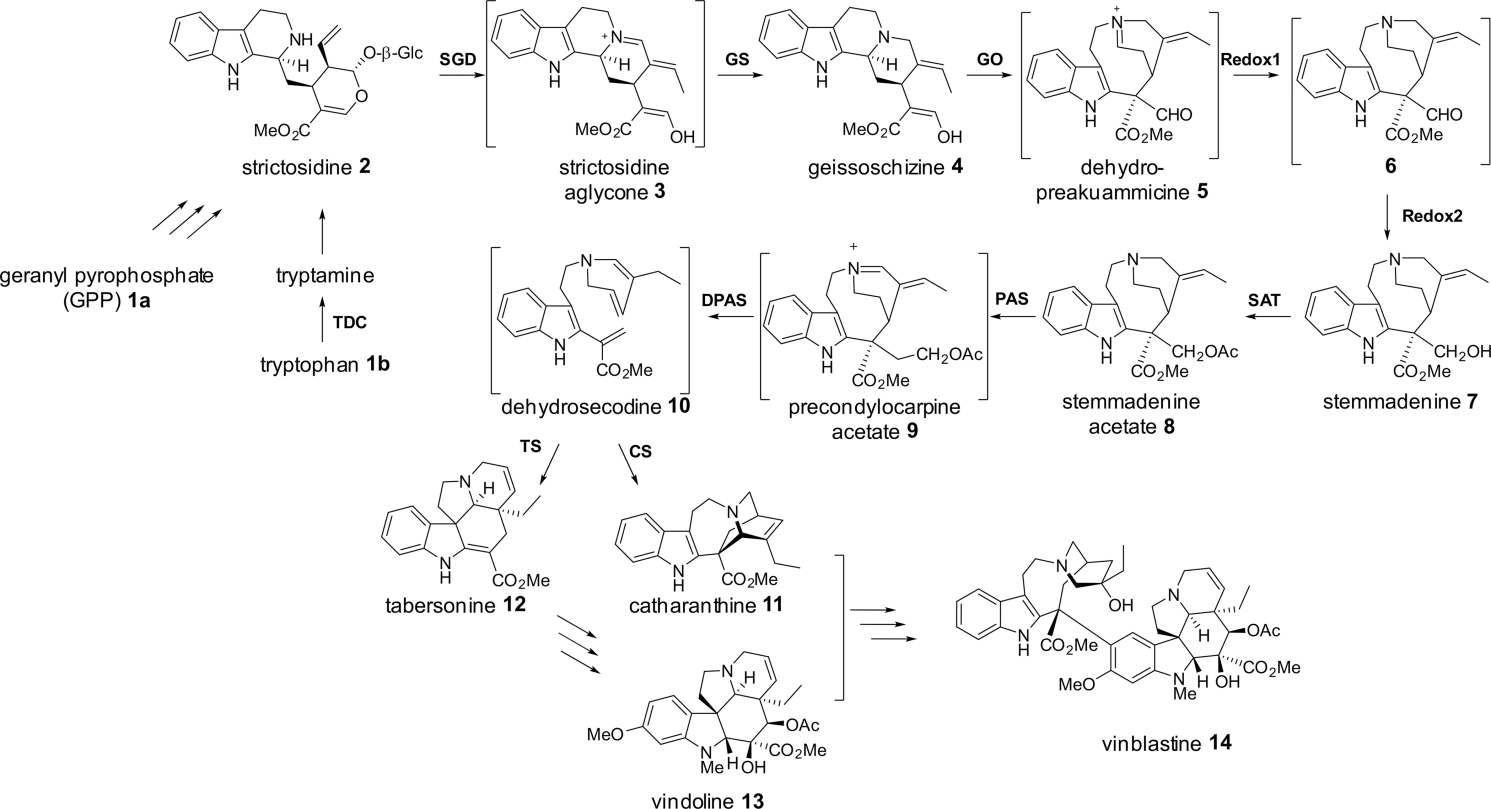
The vinblastine biosynthetic pathway. Enzymes catalyzing the steps of tabersonine and catharanthine biosynthesis from the central intermediate strictosidine are labeled in bold. Unstable intermediates are shown in square brackets. SGD, strictosidine glucosidase; GS, geissoschizine synthase; GO, geissoschizine oxidase; Redox1, Redox2; SAT, stemmadenine acetyltransferase; PAS, precondylocarpine acetate synthase; DPAS, dihydroprecondylocarpine synthase; CS, catharanthine synthase; TS, tabersonine synthase.

Vinblastine is a high-value natural product of the monoterpene indole alkaloid (MIA) class produced by the plant *Catharanthus roseus* (Figure 1). Vinblastine (**14**), along with its derivatives, are used in chemotherapy treatments for various types of cancer.^1^ Due to the low abundance of vinblastine in *C. roseus*, sourcing this molecule in amounts sufficient for pharmaceutical use is challenging.^2^ Currently, it is obtained through isolation, synthetic strategies, or semisynthetic approaches, but each of these production methods have limitations.^3^ Vinblastine (**14**) is the dimerization product of two monomers, catharanthine (**11**) and vindoline (**13**).^4^ Both of these compounds are derived from the central MIA intermediate strictosidine (**2**). Catharanthine (**11**) is formed via nine enzymatic steps from strictosidine. Production of vindoline (**13**) goes through the biosynthetic intermediate tabersonine (**12**), which has a biosynthetic pathway nearly identical with that of catharanthine.^5−7^ Recently, all of the genes responsible for the biosynthesis of catharanthine, tabersonine, and vindoline have been discovered, which allows the possibility for state-of-art synthetic biology approaches to access these complex molecules (Figure 1).^5−7^

The metabolic engineering of high value natural products in the native producer plants is often challenging, due to demanding propagation conditions, low gene transformation efficiencies, complex genetic regulation, and cross-talk between the engineered and native biosynthetic pathways.^8,9^ As an alternative strategy, biosynthetic pathways of high-value plant natural products, such as benzylisoquinoline and tropane alkaloids or diterpenes such as taxol precursors, have been reconstituted in microbial heterologous hosts.^10^ Recently, the biosynthesis of the vinblastine precursors vindoline and catharanthine has been reconstituted in *Saccharomyces cerevisiae* to produce microgram per liter titers after extensive optimization.^11^ However, transfer of a plant biosynthetic pathway to a microbial host often results in low titers, likely due to the different physiology between the producing and the host cells, and requires substantial and time-consuming optimization. Plant systems have been successfully used as an alternative production host, as model plant hosts share the cellular compartmentalization and enzyme cofactors with native plant natural product producers.^12^ Due to the ease of cultivation and genetic manipulation, *Nicotiana benthamiana* is widely used in biosynthetic pathway reconstitutions.^12,13^ Various tools have been developed for both stable and transient transformation of the *N. benthamiana* plants.^14−16^*Agrobacterium*-mediated transient gene expression in *N. benthamiana* is a widely used, rapid and efficient technique allowing coexpression of multiple transgenes to produce higher levels of desired compounds.^17^

Engineering heterologous hosts for production of high titers of MIAs is particularly difficult. The vinblastine pathway is composed of many enzymes (31 from geranyl pyrophosphate **1a** and tryptophan **1b**) and involves numerous unstable and toxic biosynthetic intermediates (**3**, **5**, **6**, **9**, **10**) (Figure 1).^5,6,18^ Some of the biosynthetic reaction products can nonenzymatically degrade to form dead-end products.^6,19^ Additionally, the pathway genes are expressed in various tissues, cell types, and subcellular compartments.^20,21^ Heterologous production of the MIA early intermediate strictosidine (**2**) has been demonstrated in both microbial and plant chassis.^18,22,23^ However, production of MIAs downstream of strictosidine is particularly problematic due to the high reactivity of numerous downstream intermediates, though successful reconstitution in yeast has been recently achieved.^7,24−26,11^ We wanted to explore the prospect of reconstitution of late stage MIA alkaloids from strictosidine in *N. benthamiana*. Here we optimize the yields of late vinblastine precursors precondylocarpine acetate (**9**), tabersonine (**12**), and catharanthine (**11**), using a modular *Agrobacterium*-mediated transient gene expression-based reconstitution approach in *N. benthamiana*. First, we investigated the effects of expressing the *C. roseus* biosynthetic genes for the intermediate stemmadenine acetate (**8**) (Figure 1) under regulatory elements of varying strength. Based on the findings, we generated two sets of multitranscriptional unit (multi-TU) vectors using different regulatory element combinations to test their efficiency in biosynthetic gene expression. Notably, instead of stemmadenine acetate (**8**), we obtained the oxidized product of this compound, precondylocarpine acetate (**9**), at yields of approximately 2.5 mg per g of fresh *N. benthamiana* tissue, suggesting that there is an endogenous *N. benthamiana* oxidase that can efficiently catalyze this reaction. Precondylocarpine acetate (**9**) is also a biosynthetic intermediate in the vinblastine biosynthetic pathway, so this endogenous activity was a fortuitous occurrence. We then successfully reconstituted the biosynthesis of tabersonine (**12**) and catharanthine (**11**) starting from the central MIA intermediate strictosidine (**2**) with titers of ca. 10 and 60 ng per g of frozen tissue, respectively. This study sets the foundation for production of the vinblastine precursors in non-native plant chassis.

## Results and Discussion

Catharanthine (**11**) and tabersonine (**12**) are produced from strictosidine (**2**) through a series of nine enzyme-catalyzed steps (Figure 1).^5,6,19^ The products (**5** and **6**) of biosynthetic steps catalyzed by the enzymes GO and Redox1 are highly unstable and therefore do not accumulate in plant extracts. This is also the case for the labile downstream intermediate dehydrosecodine (**10**).^5,6^ Both stemmadenine and stemmadenine acetate are stable intermediates, so we therefore focused on optimizing expression of the stemmadenine acetate (**8**) portion of the pathway as a first milestone.

We used *Agrobacterium*-mediated transient expression, in which separate bacterial strains each transformed with one pathway gene, were mixed, and then were transformed into *N. benthamiana*. Each vector harbored one of the six stemmadenine acetate biosynthetic enzyme genes from *C. roseus* (SGD, GS, GO, Redox1, Redox2, SAT; Figure 1). *N. benthamiana* leaves were transfected with either six *Agrobacterium* strains with all 6 biosynthetic genes under the control of the strong SlUbq10 promoter and terminator pair, or with all 6 biosynthetic genes each under the control of the weak AtuNos promoter and terminator pair to assess whether the overall strength of biosynthetic gene expression would affect the metabolite yield.^27,28^ We used the Golden Braid assembly system, which allowed us to quickly build the constructs using a suite of standardized genetic parts.^29^ To avoid the deleterious effect of RNA silencing commonly triggered in *Agrobacterium*-mediated overexpression, we also coinfiltrated an *Agrobacterium* strain harboring the P19 silencing suppressor.^30^ After 2 days, leaves were infiltrated with 1 mL of 200 μM strictosidine substrate in aqueous buffer, and harvested for mass spectrometry analysis 2 days later. While analyzing all of the assay products, we did not observe stemmadenine acetate (**8**), but instead, we observed precondylocarpine acetate (PCA, **9**), which is the oxidized product of stemmadenine acetate (Supplementary Figure S1). In vinblastine biosynthesis in *C. roseus* stemmadenine acetate is oxidized by the berberine bridge-like (BBE) enzyme PAS to form PCA (**9**) (Figure 1).^6^ Since we did not include PAS in these transient expression experiments, stemmadenine acetate must be oxidized by an endogenous enzyme in *N. benthamiana*, which is consistent with the results of previous *in planta* enzyme assays.^6^ The *N. benthamiana* genome codes for numerous BBE-like enzymes and other oxidases that could potentially harbor this nonspecific catalytic activity.^31^ As we consistently observed full conversion of stemmadenine acetate (**8**) into PCA (**9**) in all transient expression assays, we used PCA (**9**) as our target metabolite for quantification.

**Figure 2:**
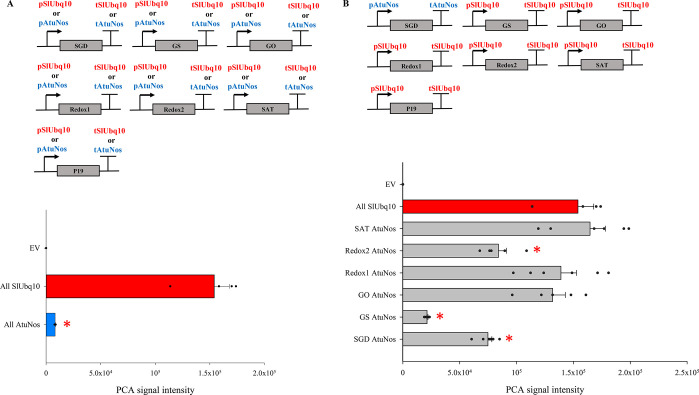
The effects of swapping regulatory elements driving the expression of each of the stemmadenine acetate biosynthetic enzyme genes on the yield of precondylocarpine acetate in *N. benthamiana* transient expression assays. (A) Combinations of single transcriptional unit constructs used in the coinfiltration assays. The average LC-MS ion abundance of precondylocarpine acetate (**9**, *m*/*z* 395.19 ± 0.05) produced in *N. benthamiana* in a sample size of 3–4 biological replicates ± SE for expression on all strong (red) and all weak (blue) regulatory elements is shown. (B) A representative combination of single transcriptional unit construct in which the SGD regulatory element is swapped for the weak (blue) pair. The average LC-MS ion abundance of precondylocarpine acetate (**9**, *m*/*z* 395.19 ± 0.05) produced in *N. benthamiana* in a sample size of 3–6 biological replicates ± SE for each of these promoter/terminator swaps. The experiment was repeated twice with similar results. The sample groups for which the average ion abundance of PCA was significantly different to that of the All SlUbq10 promoter/terminator combination are labeled with a red asterisk (Mann–Whitney U test; *p* ≤ 0.05).

We observed successful production of PCA (**9**) in the coinfiltration experiments using both the strong SlUbq10 promoter/terminator pairs and the weak AtuNos regulatory element pairs (Figure 2A). However, the coinfiltration assay where expression of all constructs was driven by the AtuNos pair resulted in an almost 16-fold lower amount of PCA than the coinfiltration assay in which all genes were placed under the control of SlUbq10 promoter/terminator constructs. This indicated that strong expression of at least some of the biosynthetic genes is required for optimal PCA production.

We set out to identify which of the six enzymes needed to be on a strong promoter/terminator pair to maintain high levels of PCA production. We designed a series of coinfiltration assays in which we sequentially swapped the strong SlUbq10 promoter/terminator pair attached to each of the six biosynthetic genes for the weak AtuNos pair (Figure 2B).^28^ Notably, when SGD, GS, and Redox2 were placed under the control of the weak AtuNos pair, PCA (**9**) titers dropped significantly (Figure 2B). In contrast, swapping a strong promoter/terminator pair for GO, Redox1 and SAT had no substantial effect on PCA titer.

In assays where SGD expression was driven by the AtuNos regulatory elements, higher amounts of unreacted strictosidine (**2**) were detected (Supplementary Figure S2), suggesting that strong expression of SGD may be needed to provide maximal levels of starting material for this branch of the metabolic pathway. Strictosidine aglycone (**3**) is a cross-linking agent that is universally toxic.^32^ GS reduces strictosidine aglycone (**3**) to form geissoschizine (**4**), which is substantially less reactive.^19,33^ The significant decrease in PCA (**9**) yield when GS is under the control of a weak promoter/terminator suggests that a strong promoter for this reductase is crucial for rapid reduction of the reactive strictosidine aglycone (**3**) intermediate before it cross-links with cell components.^33^

The enzyme reactions catalyzed by GO, Redox1, and Redox2 have been speculated to occur in a highly coregulated fashion, since the biosynthetic intermediates (**5**, **6**) produced by GO and Redox1 are chemically unstable.^5^ GO converts geissoschizine, which is a stable compound that does not degrade readily in *in vitro* assays,^19^ to a short-lived intermediate **5** that serves as a the substrate for Redox1.^5^ However, we observed no decrease in the yields of PCA (**9**) when either GO and Redox1 were placed under the control of a weak promoter/terminator. This suggested, surprisingly, that regulation of these two enzymes does not have to be matched. In the case of Redox2, which catalyzes the formation of stemmadenine (**7**) from a highly unstable intermediate (**6**) generated by Redox1,^5^ placing it under the AtuNos weak regulatory element pair caused a significant decrease in the PCA yield. Finally, SAT, an *O*-acetyltransferase, converts stemmadenine (**7**) to stemmadenine acetate (**8**), which is a relatively stable pathway intermediate. Expressing SAT under the control of AtuNos promoter/terminator pair did not cause a decrease in PCA yields.^5^ In short, we determined that for optimal titers, it was essential to have the first two genes, SGD and GS, as well as Redox2, under the control of a strong promoter. In contrast, for GO, Redox1, and SAT, the strength of the promoter/terminator pair did not appear to significantly affect product titers. The optimal promoter/terminator pair did not appear to correlate with the position of the gene in the biosynthetic pathway, the type of enzymatic transformation that the gene product catalyzed, or the chemical stability of the enzyme substrate or product. However, placing GS under the control of a weak promoter/terminator affected the yields most adversely, likely due to the high toxicity of the GS substrate. Notably, we could not detect chromatographic peaks corresponding to any of the known intermediates downstream of strictosidine in any of these transient expression experiments. *N. benthamiana* is known to derivatize a variety of compounds through glycosylation, acylation and other derivatizations.^34^ Therefore, any accumulated intermediates were likely derivatized by the native plant enzymes and therefore could not be identified using authentic compound standards available.

Using the approach described above, when many genes are required for pathway expression, this necessarily means than a correspondingly large number of *Agrobacterium* strains must be coinfiltrated into the *N. benthamiana* leaf. A large number of strains may not be efficiently transformed into all *N. benthamiana* cells, which would negatively impact product yields.^14,35^ Developments in modular cloning technologies now allow stacking of multiple transcriptional units onto a single plasmid.^29,36,37^ Attempting to improve the yield of our MIA products, we used the Golden Braid modular assembly technology to express several of the *C. roseus* biosynthetic enzymes from a single vector. A vast toolkit of regulatory elements for use in plant synthetic biology was developed in the recent years.^29,36^ However, only a few studies where multi-TU assemblies containing different regulatory elements were used for *in planta* pathway reconstitution have been reported.^38,39^

**Figure 3:**
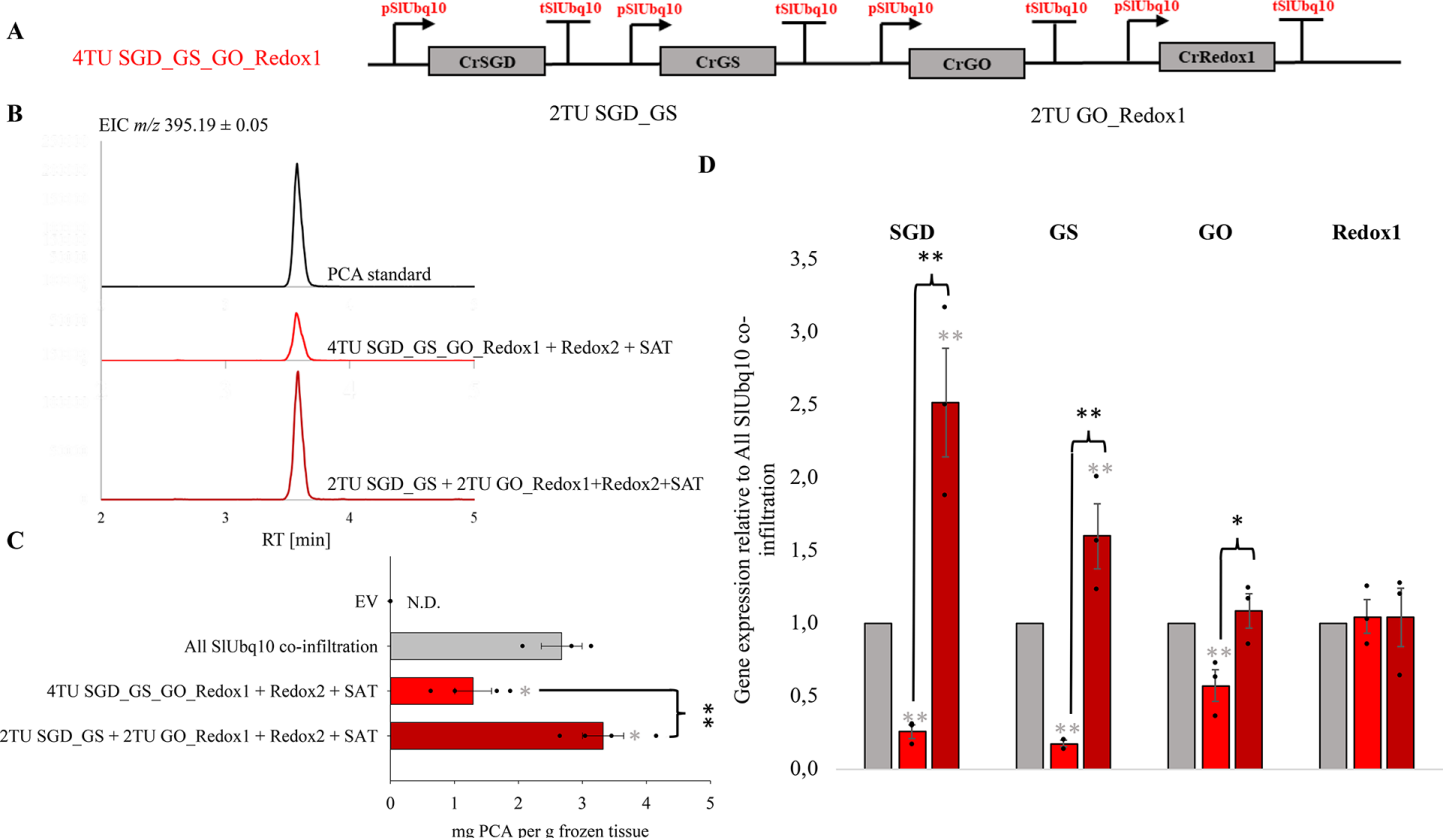
The yield of precondylocarpine acetate in transient expression experiments using the 4TU_SGD_GS_GO_Redox1 four-transcriptional unit construct in combination with Redox2 and SAT coinfiltrated on separate vectors. (A) Schematic representation of the multitranscriptional unit constructs used in the experiment with respective promoter/terminator combinations labeled. (B) LC-MS analysis of the transient expression assay products (extracted ion chromatogram *m*/*z* 395.19 ± 0.05, corresponding to precondylocarpine acetate (PCA)). (C) The average yield of precondylocarpine acetate, PCA (**9**) ± SE resulting from transient expression of multitranscriptional unit constructs coinfiltrated with downstream Redox2, SAT and P19 in *N. benthamiana* (*n* = 3–4 biological replicates). The experiment was repeated twice with similar results. The statistically significant differences between PCA (**9**) yields are marked with an asterisk. Statistically significant differences between the control All SlUbq10 coinfiltration levels and those of multigene constructs are marked with a gray asterisk (independent samples *t* test, *p* < 0.05). (D) The expression levels (±SE) of SGD, GS, GO, and Redox1 for the different multi-TU modules, relative to the levels in All SlUbq10 coinfiltration experiments (*n* = 3 biological replicates). The expression levels were compared using the 2^–ΔΔ*Ct*^ method. Statistically significant differences in gene expression between the All SlUbq10 coinfiltration control and the different multigene construct are marked with a gray asterisk (*p < 0.05; **p < 0.005, independent samples *t* test). The statistically significant differences between other sample groups are marked with black asterisks.

We built a multitranscriptional unit vector where four biosynthetic genes were expressed under the control of the SlUbq10 promoter/terminator pair (4TU_SGD_GS_GO_Redox1, Figure 3A), as this allowed rapid and efficient multi-TU assembly, despite the size and repetitiveness of the assembled sequences. To quantify the final product (PCA, **9**), we coinfiltrated the two missing biosynthetic enzymes, Redox2 and SAT, on separate vectors, each also under the control of the SlUbq10 promoter/terminator pair along with this 4TU construct. However, this experiment yielded significantly lower amounts of PCA (**9**) compared to the positive control, in which all genes were expressed on separate vectors (All SlUbq10 coinfiltration) (Figure 3B, 3C). We then tested the intermediate vectors 2TU_SGD_GS and 2TU_GO_Redox1 in which only two genes under the control of the SlUbq10 promoter/terminator pair are located on a single vector (Figure 3A). Using these 2TU vectors, along with Redox2 and SAT on separate vectors, we recovered the high levels of PCA observed when all biosynthetic genes were expressed on separate vectors (All SlUbq10 coinfiltration control), though titers were not increased compared to All SlUbq10 coinfiltration (Figure 3C).

We could not observe an accumulation of any identifiable pathway intermediates and thus were not able to pinpoint the bottleneck step of the pathway through mass spectrometry analysis of metabolites. qPCR analysis of transformed *N. benthamiana* showed a significant decrease in the relative gene expression levels of the first 3 transcriptional unit genes (SGD, GS, GO) when the 4TU_SGD_GS_GO_Redox1 vector was used, compared to the All SlUbq10 coinfiltration control (ca. 3-fold decrease for SGD, 4-fold decrease for GS, 1-fold decrease for GO) (Figure 3D). The repetitive use of the strong promoter may have caused homology-mediated epigenetic silencing or disturbed the transcriptional dynamics, when using the 4TU_SGD_GS_GO_Redox1 vector.^40,41^ This is the likely cause of the decrease in PCA yield, as the levels of Redox2 and SAT coinfiltrated on separate vectors remained high under all conditions (Supplementary Figure S3). Notably, in the 2TU_SGD_GS + 2TU_GO_Redox1 coinfiltration experiments, the relative gene expression of SGD, GS, and GO was significantly higher compared to levels of these same genes in not only the 4 TU construct 4TU SGD_GS_GO_Redox1, but also with the positive control in which all genes were expressed from separate plasmids (All SlUbq10 coinfiltration) (Figure 3D). High PCA yields were achieved with the use of both 2TU vectors, as well as assays where one 2TU construct was used and the rest of the genes were coinfiltrated on separate vectors (Supplementary Figures S4, S5).

**Figure 4:**
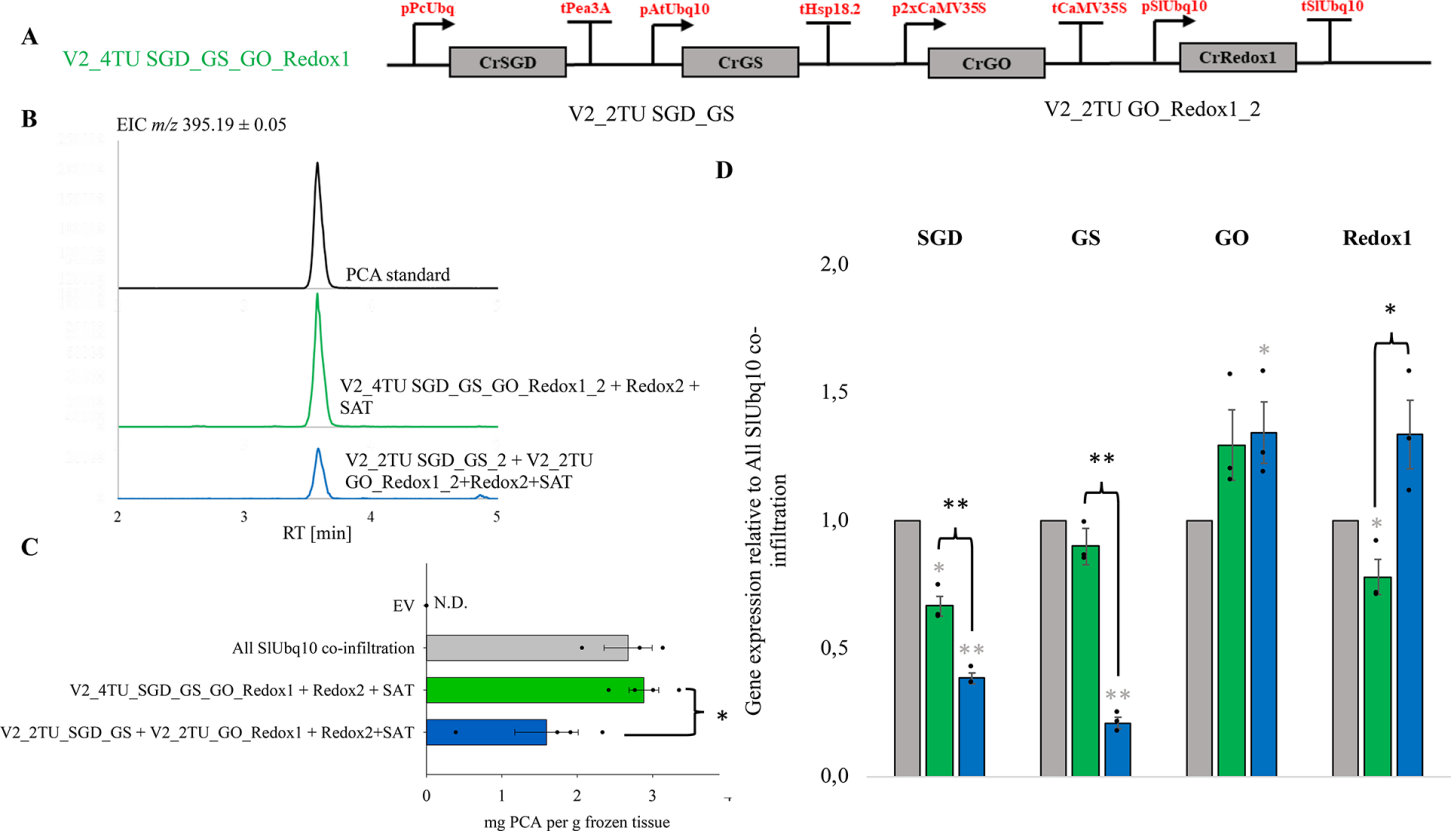
The yield of precondylocarpine acetate in transient expression experiments using the V2_4TU_SGD_GS_GO_Redox1 multitranscriptional construct in combination with Redox2 and SAT coinfiltrated on separate vectors. (A) Schematic representation of the multitranscriptional unit constructs used in the experiment with respective promoter/terminator combinations labeled. (B) LC-MS analysis of the transient expression assay products (extracted ion chromatogram, *m*/*z* 395.19 ± 0.05, corresponding to precondylocarpine acetate (PCA)). (C) The average yield of precondylocarpine acetate (PCA) ± SE resulting from transient expression of multitranscriptional unit constructs coinfiltrated with downstream Redox2, SAT, and P19 in *N. benthamiana* (*n* = 3–4 biological replicates). The experiment was repeated twice with similar results. The statistically significant difference between PCA yields are marked with an asterisk. Statistically significant differences between the control All SlUbq10 coinfiltration levels and those of multigene constructs are marked with a gray asterisk (independent samples *t* test, *p* < 0.05). (D) The expression levels (±SE) of SGD, GS, GO, and Redox1 for the different multi-TU modules, relative to the levels in All SlUbq10 coinfiltration experiments (*n* = 3 biological replicates). The expression levels were compared using the 2^–ΔΔ*Ct*^ method. Statistically significant differences in gene expression between the control All SlUbq10 and the different multigene construct are marked with a gray asterisk (*p < 0.05; **p < 0.005, independent samples *t* test). The statistically significant differences between other sample groups are marked with black asterisks.

Since the decreased expression levels of the biosynthetic genes likely resulted from the repeated use of the SlUbq10 promoter/terminator pair, we constructed a 4 TU vector containing no repetitive regulatory sequences (V2_4TU_SGD_GS_GO_Redox1) (Figure 4A). We identified 3 additional regulatory elements described in the literature reported to drive high gene expression (PcUbq10, AtUbq10, 2× CaMV35S).^42,36,37^ When V2_4TU_SGD_GS_GO_Redox1 was infiltrated, along with Redox2 and SAT on individual plasmids, with strictosidine into *N. benthamiana*, yields of PCA were similar to those obtained in the All SlUbq10 coinfiltration assays (Figure 4C). Notably, although SGD and Redox1 both showed a significant but modest decrease in the relative gene expression (0.7- and 0.8-fold difference, respectively) when this V2_4TU_SGD_GS_GO_Redox1 construct was used, the overall average PCA yield did not appear to be substantially decreased (Figure 4D, Supplementary Figure S6).

**Figure 5:**
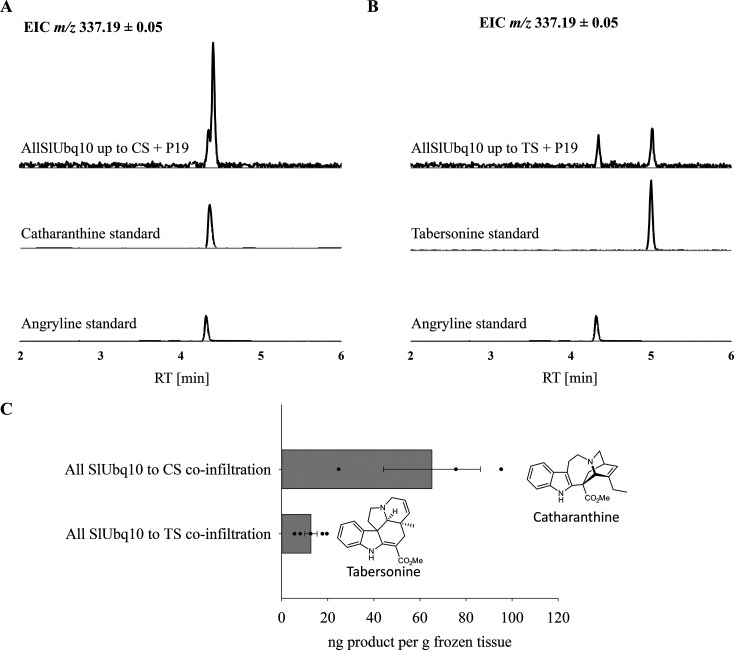
Production of catharanthine and tabersonine from transient expression in *N. benthamiana.* The extracted ion chromatograms (*m*/*z* 337.19 ± 0.05, corresponding to the mass of catharanthine, tabersonine, and the intermediate angryline) of transiently expressed products. The catharanthine/tabersonine pathway enzyme genes were coinfiltrated on separate vectors (SGD, GS, GO, Redox1, Redox2, SAT, DPAS, PAS, CS, or TS). (A) LC-MS analysis of the catharanthine pathway enzyme assays. (B) LC-MS analysis of the tabersonine pathway enzyme assays. Residual angryline (substrate of CS and TS) was detected in both assays. MS/MS spectra of the assay products are shown in Supplementary Figures S7 and S8. (C) Quantification of tabersonine and catharanthine production.

We then set out to reconstitute the more complex downstream alkaloids tabersonine (**12**) and catharanthine (**11**), which are derived from PCA (**9**). Since stacking 4 transcriptional units on a single vector did not lead to a significant increase in PCA (**9**) yields (Figure 4C), we reconstituted the tabersonine (**12**) and catharanthine (**11**) pathway by coinfiltrating *Agrobacterium* strains, with each harboring one of the following biosynthetic genes, SGD, GS, GO, Redox1, Redox2, and SAT, along with the downstream PAS, DPAS, and either CS or TS, where each gene was cloned into a vector with the SlUbq10 promoter and terminator pair individually transformed into separate *A. tumefaciens* strains. PAS was included even though an endogenous enzyme with this same activity was present. These 9 strains alongside the P19 construct carrying strain were then mixed in equal proportions, and infiltrated into *N. benthamiana.* We observed production of both catharanthine (**11**) and tabersonine (**12**) at levels of approximately 60 and 10 ng of product per g of frozen *N. benthamiana* tissue, respectively (Figure 5A,B).

The rapid development of plant biotechnology tools in recent years creates new opportunities to reconstitute natural product biosynthetic pathways in plant-based heterologous hosts.^43^ Here, we successfully reconstituted the biosynthesis of the late vinblastine precursor precondylocarpine acetate (**9**) (∼2.7 mg per g frozen tissue) using transient expression in *N. benthamiana* leaves starting from the early intermediate strictosidine (**2**). We found that three of the six stemmadenine acetate biosynthetic genes (SGD, GS, Redox2) need to be under the control of strong promoter/terminator pairs to achieve the highest product yields. Placement of GS under the control of a weak promoter/terminator led to the most dramatic decreases in titer, presumably due to the toxic nature of the GS substrate. We showed that expressing four biosynthetic enzyme genes on a single construct, instead of individual constructs, in *Agrobacterium-*mediated transient expression assays does not significantly increase the yield of the final product. We also observed the negative effect of using repeated regulatory sequences in the multi-TU assemblies that could be eliminated when different regulatory element combinations were used. Finally, we successfully reconstituted the production of the vinblastine precursors tabersonine (**12**) and catharanthine (**11**), starting from strictosidine (**2**). *N. benthamiana* is a convenient and rapid system for the production of complex, lengthy pathways that encode production of plant natural products.

## Supporting Information Available

Supplementary LC-MS data, results of relative expression level analysis of the transiently expressed genes, schemes outlining the design and assembly of the multitranscriptional unit constructs, table of the genetic elements used for assembly of the multigene constructs, sequences of primers used in qPCR analysis and plasmid assembly confirmation, parameters used for metabolite detection using the TQS, methods of construction of the multitranscriptional unit vectors, synthesis of strictosidine, *Agrobacterium*-mediated transient expression in *N. benthamiana*, UHPLC-MS analysis of the transient expression assays products, qPCR analysis of the *N. benthamiana* samples (PDF)

## References

[ref1] Dhyani P.; Quispe C.; Sharma E.; Bahukhandi A.; Sati P.; Attri D. C.; Szopa A.; Sharifi-Rad J.; Docea A. O.; Mardare I.; Calina D.; Cho W. C. Anticancer potential of alkaloids: a key emphasis to colchicine, vinblastine, vincristine, vindesine, vinorelbine and vincamine. Cancer Cell Int. 2022, 22, 206 10.1186/s12935-022-02624-9. 35655306 PMC9161525

[ref2] Sharma A.; Amin D.; Sankaranarayanan A.; Arora R.; Mathur A. K. Present status of Catharanthus roseus monoterpenoid indole alkaloids engineering in homo- and hetero-logous systems. Biotechnol. Lett. 2020, 42, 11–23. 10.1007/s10529-019-02757-4. 31729591

[ref3] Verma A.; Laakso I.; Seppänen-Laakso T.; Huhtikangas A.; Riekkola M.-L. A simplified procedure for indole alkaloid extraction from Catharanthus roseus combined with a semi-synthetic production process for vinblastine. Molecules 2007, 12, 1307–1315. 10.3390/12071307. 17909486 PMC6149338

[ref4] O’Connor S. E.; Maresh J. J. Chemistry and biology of monoterpene indole alkaloid biosynthesis. Nat. Prod Rep 2006, 23, 532–547. 10.1039/b512615k. 16874388

[ref5] Qu Y.; Easson M. E. A. M.; Simionescu R.; Hajicek J.; Thamm A. M. K.; Salim V.; De Luca V. Solution of the multistep pathway for assembly of corynanthean, strychnos, iboga, and aspidosperma monoterpenoid indole alkaloids from 19 *E* -geissoschizine. Proc. Natl. Acad. Sci. U. S. A. 2018, 115, 3180–3185. 10.1073/pnas.1719979115. 29511102 PMC5866588

[ref6] Caputi L.; Franke J.; Farrow S. C.; Chung K.; Payne R. M. E.; Nguyen T.-D.; Dang T.-T. T.; Carqueijeiro I. S. T.; Koudounas K.; de Bernonville T. D.; Ameyaw B.; Jones D. M.; Vieira I. J. C.; Courdavault V.; O’Connor S. E. Missing enzymes in the biosynthesis of the anticancer drug vinblastine in Madagascar periwinkle. Science 2018, 360, 1235–1239. 10.1126/science.aat4100. 29724909

[ref7] Qu Y.; Easson M. L. A. E.; Froese J.; Simionescu R.; Hudlicky T.; De Luca V. Completion of the seven-step pathway from tabersonine to the anticancer drug precursor vindoline and its assembly in yeast. Proc. Natl. Acad. Sci. U.S.A. 2015, 112, 6224–6229. 10.1073/pnas.1501821112. 25918424 PMC4434687

[ref8] Canter P. H.; Thomas H.; Ernst E. Bringing medicinal plants into cultivation: opportunities and challenges for biotechnology. Trends Biotechnol. 2005, 23, 180–185. 10.1016/j.tibtech.2005.02.002. 15780709

[ref9] Mora-Vásquez S.; Wells-Abascal G. G.; Espinosa-Leal C.; Cardineau G. A.; García-Lara S. Application of metabolic engineering to enhance the content of alkaloids in medicinal plants. Metabolic Engineering Communications 2022, 14, e00194 10.1016/j.mec.2022.e00194. 35242556 PMC8881666

[ref10] Wang H.; Guo H.; Wang N.; Huo Y.-X. Toward the heterologous biosynthesis of plant natural products: gene discovery and characterization. ACS Synth. Biol. 2021, 10, 2784–2795. 10.1021/acssynbio.1c00315. 34757715

[ref11] Zhang J.; Hansen L. G.; Gudich O.; Viehrig K.; Lassen L. M. M.; Schrübbers L.; Adhikari K. B.; Rubaszka P.; Carrasquer-Alvarez E.; Chen L.; D’Ambrosio V.; Lehka B.; Haidar A. K.; Nallapareddy S.; Giannakou K.; Laloux M.; Arsovska D.; Jørgensen M. A. K.; Chan L. J. G.; Kristensen M.; Christensen H. B.; Sudarsan S.; Stander E. A.; Baidoo E.; Petzold C. J.; Wulff T.; O’Connor S. E.; Courdavault V.; Jensen M. K.; Keasling J. D. A microbial supply chain for production of the anti-cancer drug vinblastine. Nature 2022, 609, 341–347. 10.1038/s41586-022-05157-3. 36045295 PMC9452304

[ref12] Reed J.; Osbourn A. Engineering terpenoid production through transient expression in Nicotiana benthamiana. Plant Cell Rep 2018, 37, 1431–1441. 10.1007/s00299-018-2296-3. 29786761 PMC6153650

[ref13] Bally J.; Jung H.; Mortimer C.; Naim F.; Philips J. G.; Hellens R.; Bombarely A.; Goodin M. M.; Waterhouse P. M. The rise and rise of Nicotiana benthamiana: a plant for all reasons. Annual Review of Phytopathology 2018, 56, 405–426. 10.1146/annurev-phyto-080417-050141. 30149789

[ref14] Stephenson M. J.; Reed J.; Patron N. J.; Lomonossoff G. P.; Osbourn A. Engineering tobacco for plant natural product production. In Comprehensive Natural Products, 3rd ed.; Liu H., Begley T., Ed.; Elsevier, 2020; pp 244–262.

[ref15] Nekrasov V.; Staskawicz B.; Weigel D.; Jones J. D. G.; Kamoun S. Targeted mutagenesis in the model plant Nicotiana benthamiana using Cas9 RNA-guided endonuclease. Nat. Biotechnol. 2013, 31, 691–693. 10.1038/nbt.2655. 23929340

[ref16] Li J.-F.; Norville J. E.; Aach J.; McCormack M.; Zhang D.; Bush J.; Church G. M.; Sheen J. Multiplex and homologous recombination-mediated genome editing in Arabidopsis and Nicotiana benthamiana using guide RNA and Cas9. Nat. Biotechnol. 2013, 31, 688–691. 10.1038/nbt.2654. 23929339 PMC4078740

[ref17] Kapila J.; De Rycke R.; Van Montagu M.; Angenon G. An Agrobacterium-mediated transient gene expression system for intact leaves. Plant Science 1997, 122, 101–108. 10.1016/S0168-9452(96)04541-4.

[ref18] Miettinen K.; Dong L.; Navrot N.; Schneider T.; Burlat V.; Pollier J.; Woittiez L.; van der Krol S.; Lugan R.; Ilc T.; Verpoorte R.; Oksman-Caldentey K.-M.; Martinoia E.; Bouwmeester H.; Goossens A.; Memelink J.; Werck-Reichhart D. The seco-iridoid pathway from Catharanthus roseus. Nat. Commun. 2014, 5, 3606 10.1038/ncomms4606. 24710322 PMC3992524

[ref19] Tatsis E. C.; Carqueijeiro I.; Dugé de Bernonville T.; Franke J.; Dang T.-T. T.; Oudin A.; Lanoue A.; Lafontaine F.; Stavrinides A. K.; Clastre M.; Courdavault V.; O’Connor S. E. A three enzyme system to generate the Strychnos alkaloid scaffold from a central biosynthetic intermediate. Nat. Commun. 2017, 8, 316 10.1038/s41467-017-00154-x. 28827772 PMC5566405

[ref20] Yamamoto K.; Takahashi K.; Caputi L.; Mizuno H.; Rodriguez-Lopez C. E.; Iwasaki T.; Ishizaki K.; Fukaki H.; Ohnishi M.; Yamazaki M.; Masujima T.; O’Connor S. E.; Mimura T. The complexity of intercellular localisation of alkaloids revealed by single-cell metabolomics. New Phytologist 2019, 224, 848–859. 10.1111/nph.16138. 31436868

[ref21] Ozber N.; Watkins J. L.; Facchini P. J. Back to the plant: overcoming roadblocks to the microbial production of pharmaceutically important plant natural products. J. Ind. Microbiol Biotechnol 2020, 47, 815–828. 10.1007/s10295-020-02300-9. 32772209

[ref22] Brown S.; Clastre M.; Courdavault V.; O’Connor S. E. De novo production of the plant-derived alkaloid strictosidine in yeast. Proc. Natl. Acad. Sci. U.S.A. 2015, 112, 3205–3210. 10.1073/pnas.1423555112. 25675512 PMC4371906

[ref23] Dudley Q. M.; Jo S.; Guerrero D. A. S.; Chhetry M.; Smedley M. A.; Harwood W. A.; Sherden N. H.; O’Connor S. E.; Caputi L.; Patron N. J. Reconstitution of monoterpene indole alkaloid biosynthesis in genome engineered Nicotiana benthamiana. Commun. Biol. 2022, 5, 1–12. 10.1038/s42003-022-03904-w. 36088516 PMC9464250

[ref24] Liu T.; Huang Y.; Jiang L.; Dong C.; Gou Y.; Lian J. Efficient production of vindoline from tabersonine by metabolically engineered Saccharomyces cerevisiae. Commun. Biol. 2021, 4, 1–9. 10.1038/s42003-021-02617-w. 34531512 PMC8446080

[ref25] Misa J.; Billingsley J. M.; Niwa K.; Yu R. K.; Tang Y. Engineered production of strictosidine and analogues in yeast. ACS Synth. Biol. 2022, 11, 1639–1649. 10.1021/acssynbio.2c00037. 35294193 PMC9171786

[ref26] Liu T.; Gou Y.; Zhang B.; Gao R.; Dong C.; Qi M.; Jiang L.; Ding X.; Li C.; Lian J. Construction of ajmalicine and sanguinarine de novo biosynthetic pathways using stable integration sites in yeast. Biotechnol. Bioeng. 2022, 119, 1314–1326. 10.1002/bit.28040. 35060115

[ref27] Dahan-Meir T.; Filler-Hayut S.; Melamed-Bessudo C.; Bocobza S.; Czosnek H.; Aharoni A.; Levy A. A. Efficient in planta gene targeting in tomato using geminiviral replicons and the CRISPR/Cas9 system. Plant Journal 2018, 95, 5–16. 10.1111/tpj.13932. 29668111

[ref28] Andreou A. I.; Nirkko J.; Ochoa-Villarreal M.; Nakayama N. Mobius assembly for plant systems highlights promoter-terminator interaction in gene regulation. bioRxiv, March 31, 2021. 10.1101/2021.03.31.437819.

[ref29] Sarrion-Perdigones A.; Vazquez-Vilar M.; Palací J.; Castelijns B.; Forment J.; Ziarsolo P.; Blanca J.; Granell A.; Orzaez D. GoldenBraid 2.0: A comprehensive DNA assembly framework for plant synthetic biology. Plant Physiol. 2013, 162, 1618 10.1104/pp.113.217661. 23669743 PMC3707536

[ref30] Garabagi F.; Gilbert E.; Loos A.; McLean M. D.; Hall J. C. Utility of the P19 suppressor of gene-silencing protein for production of therapeutic antibodies in Nicotiana expression hosts. Plant Biotechnology Journal 2012, 10, 1118–1128. 10.1111/j.1467-7652.2012.00742.x. 22984968

[ref31] Kajikawa M.; Shoji T.; Kato A.; Hashimoto T. Vacuole-localized berberine bridge enzyme-like proteins are required for a late step of nicotine biosynthesis in Tobacco. Plant Physiol 2011, 155, 2010–2022. 10.1104/pp.110.170878. 21343426 PMC3091092

[ref32] Guirimand G.; Courdavault V.; Lanoue A.; Mahroug S.; Guihur A.; Blanc N.; Giglioli-Guivarc’h N.; St-Pierre B.; Burlat V. Strictosidine activation in Apocynaceae: towards a “nuclear time bomb”?. BMC Plant Biol. 2010, 10, 182 10.1186/1471-2229-10-182. 20723215 PMC3095312

[ref33] Stavrinides A.; Tatsis E. C.; Foureau E.; Caputi L.; Kellner F.; Courdavault V.; O’Connor S. E. Unlocking the Diversity of Alkaloids in Catharanthus roseus: Nuclear localization suggests metabolic channeling in secondary metabolism. Chemistry & Biology 2015, 22, 336–341. 10.1016/j.chembiol.2015.02.006. 25772467 PMC4372254

[ref34] Gülck T.; Booth J. K.; Carvalho Â.; Khakimov B.; Crocoll C.; Motawia M. S.; Møller B. L.; Bohlmann J.; Gallage N. J. Synthetic Biology of Cannabinoids and Cannabinoid Glucosides in Nicotiana benthamiana and Saccharomyces cerevisiae. J. Nat. Prod. 2020, 83, 2877–2893. 10.1021/acs.jnatprod.0c00241. 33000946

[ref35] Montague N. P.; Thuenemann E. C.; Saxena P.; Saunders K.; Lenzi P.; Lomonossoff G. P. Recent advances of Cowpea mosaic virus-based particle technology. Human Vaccines 2011, 7, 383–390. 10.4161/hv.7.3.14989. 21368585

[ref36] Engler C.; Youles M.; Gruetzner R.; Ehnert T.-M.; Werner S.; Jones J. D. G.; Patron N. J.; Marillonnet S. A Golden Gate modular cloning toolbox for plants. ACS Synth. Biol. 2014, 3, 839–843. 10.1021/sb4001504. 24933124

[ref37] Andreou A. I.; Nakayama N. Mobius Assembly: A versatile Golden-Gate framework towards universal DNA assembly. PLoS One 2018, 13, e0189892 10.1371/journal.pone.0189892. 29293531 PMC5749717

[ref38] Forestier E. C. F.; Czechowski T.; Cording A. C.; Gilday A. D.; King A. J.; Brown G. D.; Graham I. A. Developing a Nicotiana benthamiana transgenic platform for high-value diterpene production and candidate gene evaluation. Plant Biotechnol. J. 2021, 19, 1614–1623. 10.1111/pbi.13574. 33657678 PMC8384591

[ref39] Kallam K.; Moreno-Giménez E.; Mateos-Fernández R.; Tansley C.; Gianoglio S.; Orzaez D.; Patron N. J. Tunable control of insect pheromone biosynthesis in *Nicotiana benthamiana*. bioRxiv, June 15, 2022. 10.1101/2022.06.15.496242v1.full. PMC10281601 37032497

[ref40] Rajeevkumar S.; Anunanthini P.; Sathishkumar R. Epigenetic silencing in transgenic plants. Front. Plant Sci. 2015, 10.3389/fpls.2015.00693. PMC4564723 26442010

[ref41] Johnstone C. P.; Galloway K. E. Supercoiling-mediated feedback rapidly couples and tunes transcription. Cell Reports 2022, 41, 111492 10.1016/j.celrep.2022.111492. 36261020 PMC9624111

[ref42] Durr J.; Papareddy R.; Nakajima K.; Gutierrez-Marcos J. Highly efficient heritable targeted deletions of gene clusters and non-coding regulatory regions in Arabidopsis using CRISPR/Cas9. Sci. Rep 2018, 8, 4443 10.1038/s41598-018-22667-1. 29535386 PMC5849686

[ref43] Barnum C. R.; Endelman B. J.; Shih P. M. Utilizing plant synthetic biology to improve human health and wellness. Front. Plant Sci. 2021, 10.3389/fpls.2021.691462. PMC8421571 34504505

