# Supplementary material for: Engineering the Biosynthesis of Late-Stage Vinblastine Precursors Precondylocarpine Acetate, Catharanthine, Tabersonine in Nicotiana benthamiana
Source: ACS Synth Biol. 2022 Dec 14;12(1):27–34. doi: 10.1021/acssynbio.2c00434 (PMC9872167; doi:10.1021/acssynbio.2c00434)
Supplement: Supplementary file 1 — sb2c00434_si_001.pdf [file sb2c00434_si_001.pdf]

Supplementary Materials and Methods

A modular approach towards engineering the biosynthesis of vinblastine biosynthetic precursors- catharanthine and tabersonine in *Nicotiana benthamiana*

Dagny Grzech[1], Benke Hong[1], Lorenzo Caputi[1], Prashant D. Sonawane[1], Sarah E O'Connor[1*]

[1] Department of Natural Product Biosynthesis, Max Planck Institute for Chemical Ecology, Jena, Germany

*oconnor@ice.mpg.de

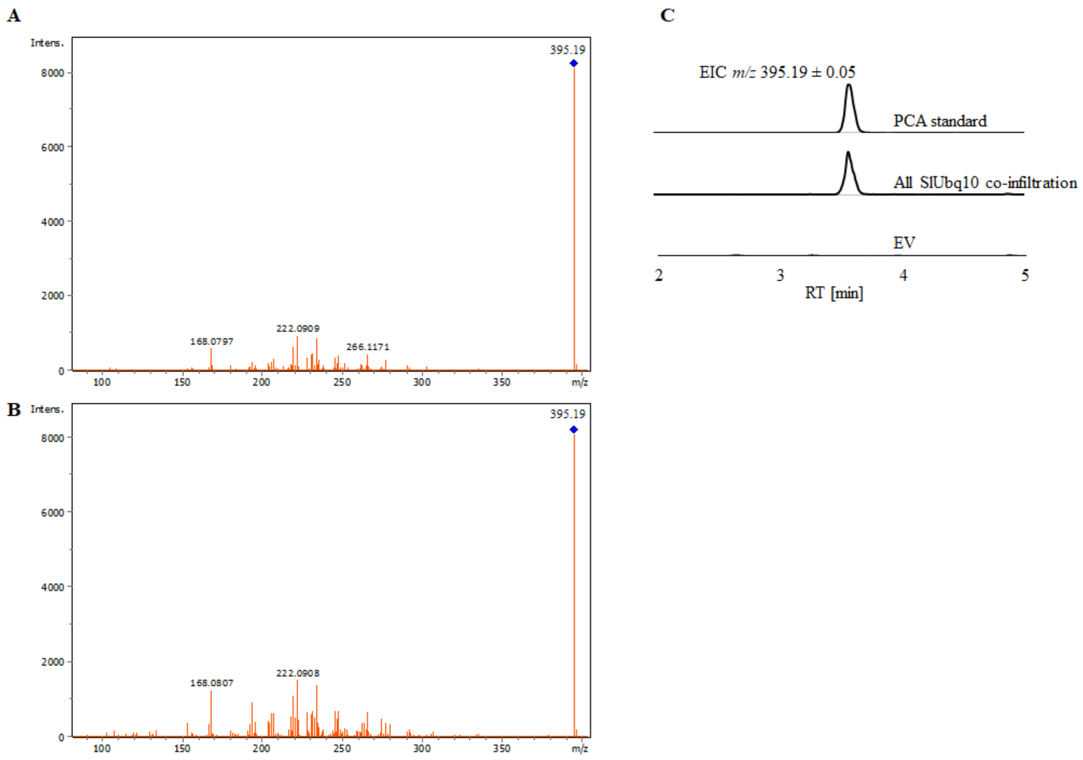

Supplementary Figure 1. Precondylocarpine acetate (PCA) detected in the extractions of transient expression assays using SGD, GS, GO, Redox1, Redox2 and SAT. A. MS/MS spectrum of a PCA standard. The precursor ion (*m/z* 395.19) is marked with a blue diamond. **B.** MS/MS spectrum of a PCA peak in the assay products. The precursor ion (*m/z* 395.19) is marked with a blue diamond. **C.** Chromatograms of the PCA standard, the extract of the transient expression assays and the empty vector (EV) control.

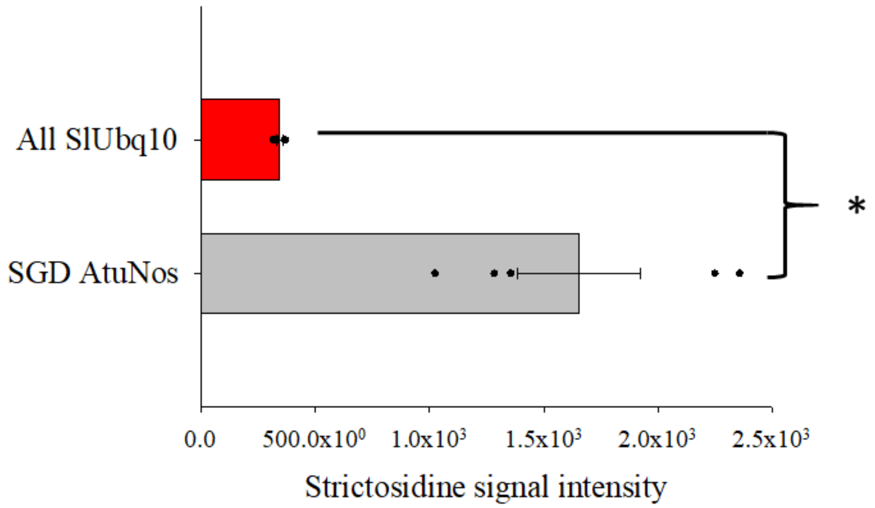

Supplementary Figure 2. Average LC-MS ion abundance of strictosidine (*m/z* 531.23 ± 0.05) in All SlUbq10 in which SGD, GS, GO, Redox1, Redox2 and SAT are all under the control of the strong SlUbq10 promoter/terminator (red) compared to the same experiment in which only the promoter/terminator of SGD is swapped with the weak promoter/terminator AtuNos (gray) in *N. bentamiana* in a sample size 4-6 biological replicates ± S.E. There was a significant difference in the ion abundance of strictosidine between the two sample groups (Mann-Whitney U test; $p \leq 0.05$).

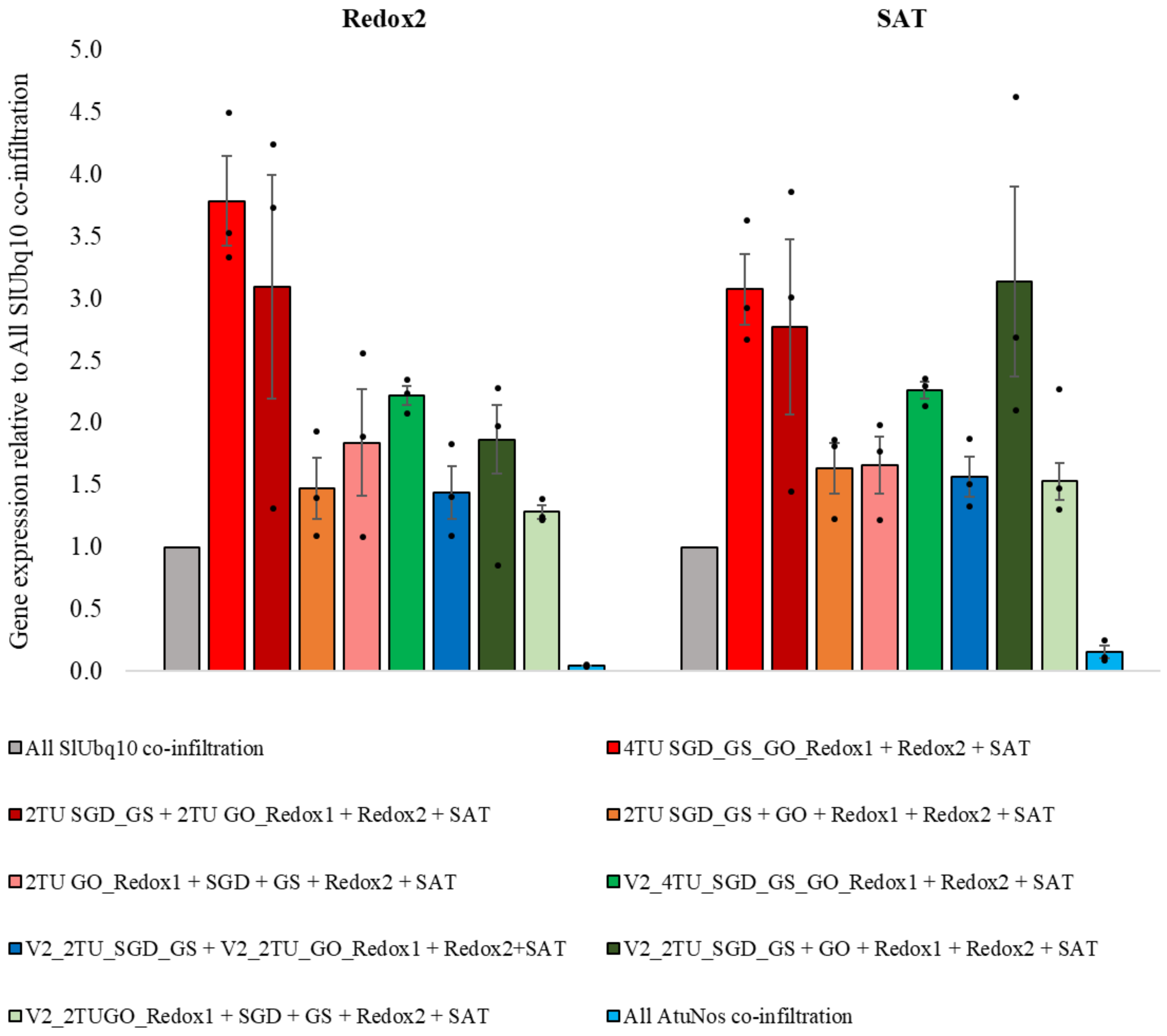

Supplementary Figure 3. The expression levels (± S.E.) of Redox2 and SAT genes, which were always infiltrated on individual plasmids, measured for the different multi-transcriptional unit modules, relative to the levels in All SlUbq10 co-infiltration experiments (n = 3 biological replicates). The expression levels were compared through qPCR, using the $2^{-\Delta\Delta Ct}$ method.

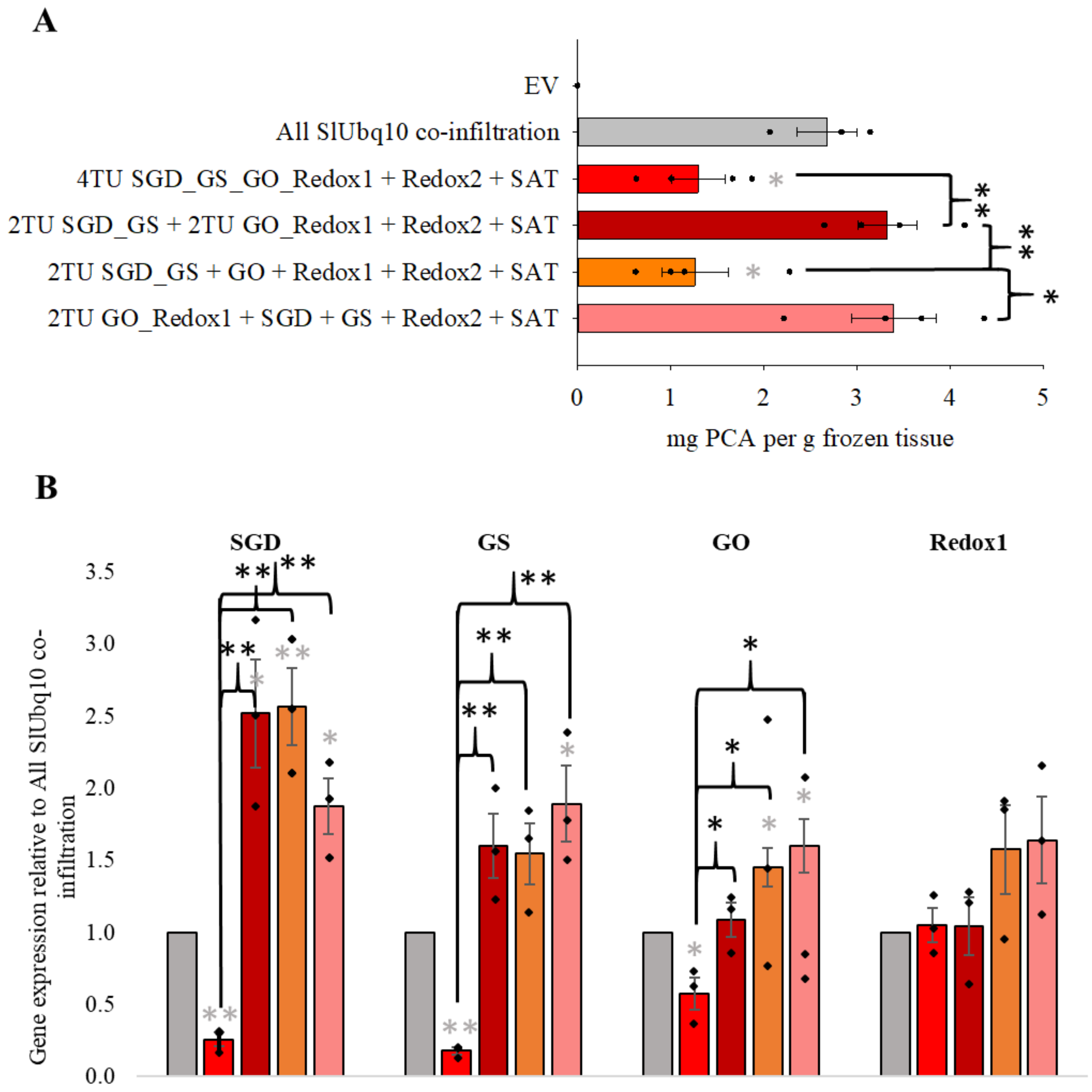

Supplementary Figure 4. The yield of precondylocarpine acetate in transient expression experiments using the 4TU_SGD_GS_GO_Redox1 multi-transcriptional construct and the intermediate constructs in combination with Redox2 and SAT co-infiltrated on separate vectors A. The average yield of precondylocarpine acetate ± S.E. resulting from transient expression of multi-transcriptional unit constructs co-infiltrated with downstream Redox2, SAT and P19 in *N. benthamiana* (n = 3- 4 biological replicates). The experiment was repeated twice with similar results. The statistically significant difference between PCA yields are marked with an asterisk (Independent samples T-test, $p < 0.05$). **B.** The expression levels (± S.E.) of the SGD, GS, GO and Redox1 measured for the different multi-transcriptional unit modules, relative to the levels in All SlUbq10 co-infiltration experiments (n = 3 biological replicates). The expression levels were compared through qPCR, using the $2^{-\Delta\Delta Ct}$ method. Statistically significant differences in gene expression between the control All SlUbq10 co-infiltration levels and levels achieved in different multi-gene construct are marked with a gray

asterisk (* p<0.05; ** p<0.005, Independent samples T-test). The statistically significant differences between other sample groups are marked with black asterisks.

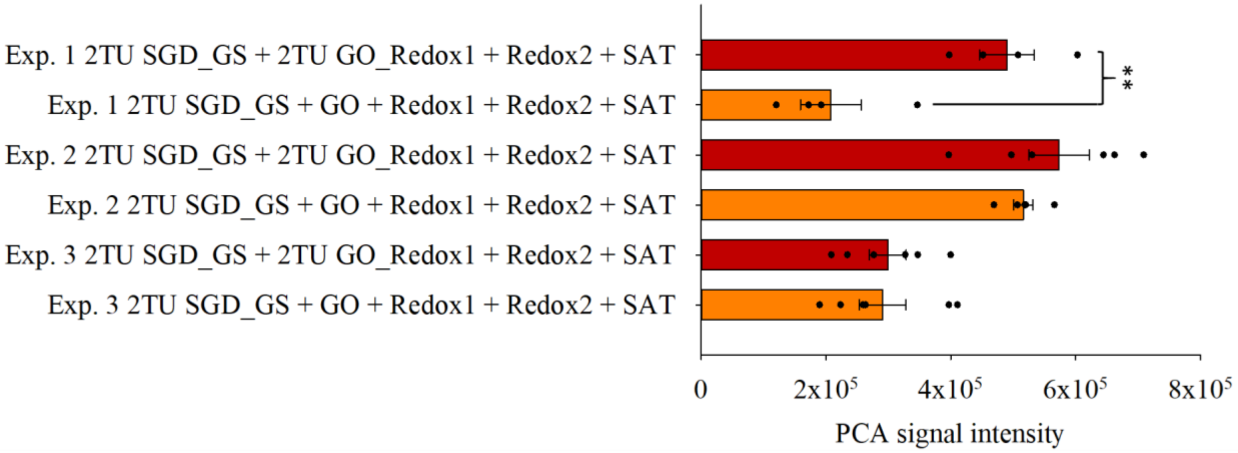

Supplementary Figure 5. Comparison three independent experiments of transient expression. The average yields of precondylocarpine acetate ± S.E. resulting from transient expression of 2TU_SGD_GS (GO, Redox1, Redox2, SAT co-infiltrated on separate vectors and 2TU_SGD_GS + 2TU_GO_Redox1 combinations (Redox2, SAT co-infiltrated on separate vectors) across 3 independent experiments. The significant difference between the two sample groups was observed only in Exp. 1 (** p<0.005, Independent samples T-test).

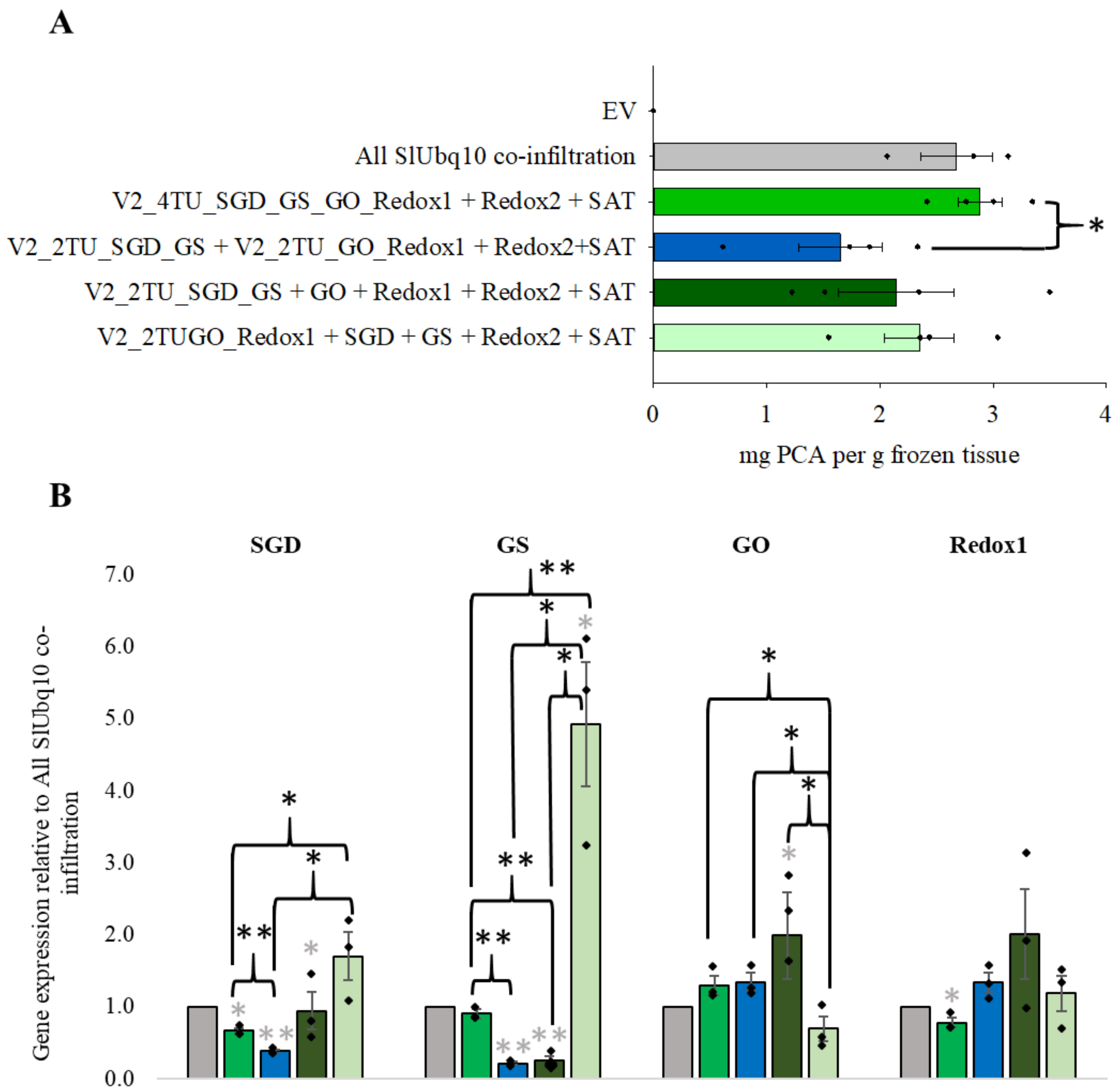

Supplementary Figure 6. The yield of precondylocarpine acetate in transient expression experiments using the V2_4TU_SGD_GS_GO_Redox1 multi-transcriptional construct and the intermediate constructs in combination with Redox2 and SAT co-infiltrated on separate vectors A. The average yield of precondylocarpine acetate ± S.E. resulting from transient expression of multi-transcriptional unit constructs co-infiltrated with downstream Redox2, SAT and P19 in *N. benthamiana* (n = 3- 4 biological replicates). The experiment was repeated twice with similar results. The statistically significant difference between PCA yields are marked with an asterisk (Independent samples T-test, $p < 0.05$). **D.** The expression levels (± S.E.) of the SGD, GS, GO and Redox1 measured for the different multi-transcriptional unit modules, relative to the levels in All SlUbq10 co-infiltration experiments (n = 3 biological replicates). The expression levels were compared through qPCR, using the $2^{-\Delta\Delta Ct}$ method. Statistically significant differences in gene expression between the control All SlUbq10 co-infiltration levels and levels achieved in different multi-gene construct are marked with a gray asterisk (* p<0.05; ** p<0.005, Independent samples T-test). The statistically significant differences between other sample groups are marked with black asterisks.

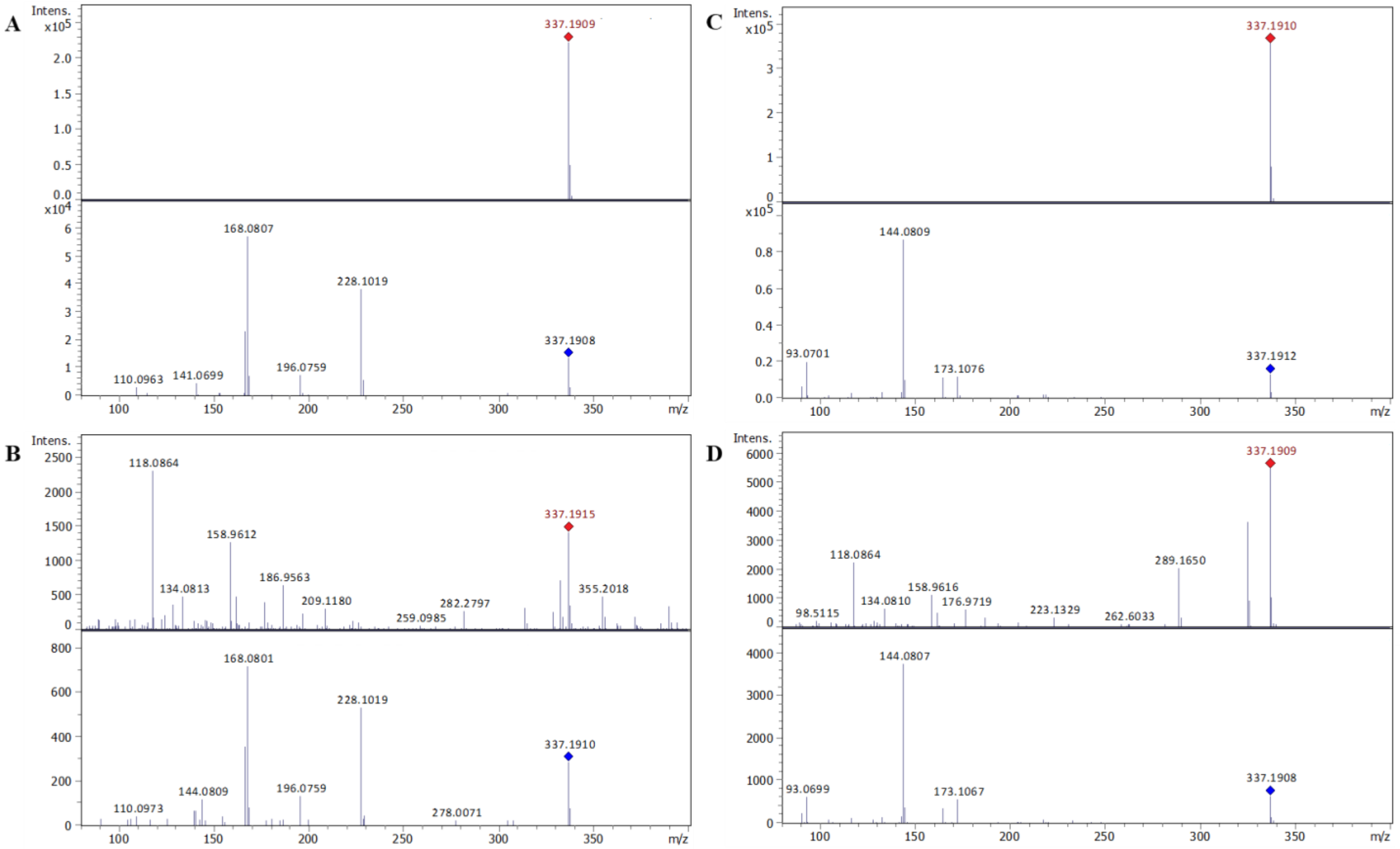

Supplementary Figure 7. MS/MS spectra of the products of the catharanthine production transient expression assays. A. Spectra of the angryline standard. The upper panel shows the angryline precursor ion. The lower panel shows the resulting fragment ions. **B.** Spectra of the angryline produced in the assay of the catharanthine biosynthetic enzymes. The upper panel shows the angryline precursor ion. The lower panel shows the resulting fragment ions. **C.** Spectra of the catharanthine standard. The upper panel shows the catharanthine precursor ion. The lower panel shows the resulting fragment ions. **D**. Spectra catharanthine produced in the assay. The upper panel shows the catharanthine precursor ion. The lower panel shows the resulting fragment ions.

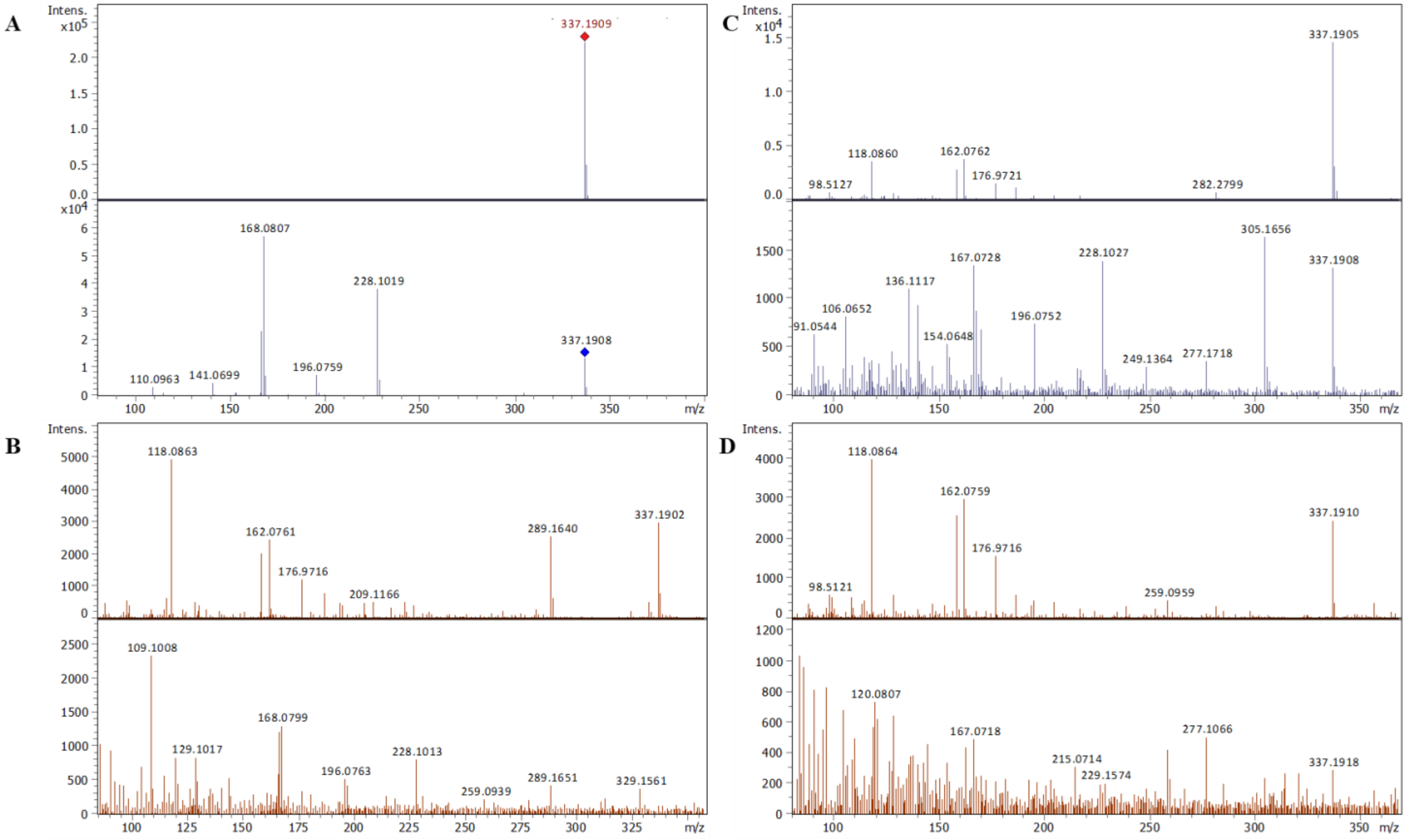

Supplementary Figure 8. MS/MS spectra of the products of the tabersonine production transient expression assays. A. Spectra of the angryline standard. The upper panel shows the angryline precursor ion. The lower panel shows the resulting fragment ions. **B.** Spectra of the angryline produced in the assay of the tabersonine biosynthetic enzymes. The upper panel shows the angryline precursor ion. The lower panel shows the resulting fragment ions. **C.** Spectra of the tabersonine standard. The upper panel shows the catharanthine precursor ion. The lower panel shows the resulting fragment ions. **D**. Spectra of tabersonine produced in the assay. The upper panel shows the tabersonine precursor ion. The lower panel shows the resulting fragment ions.

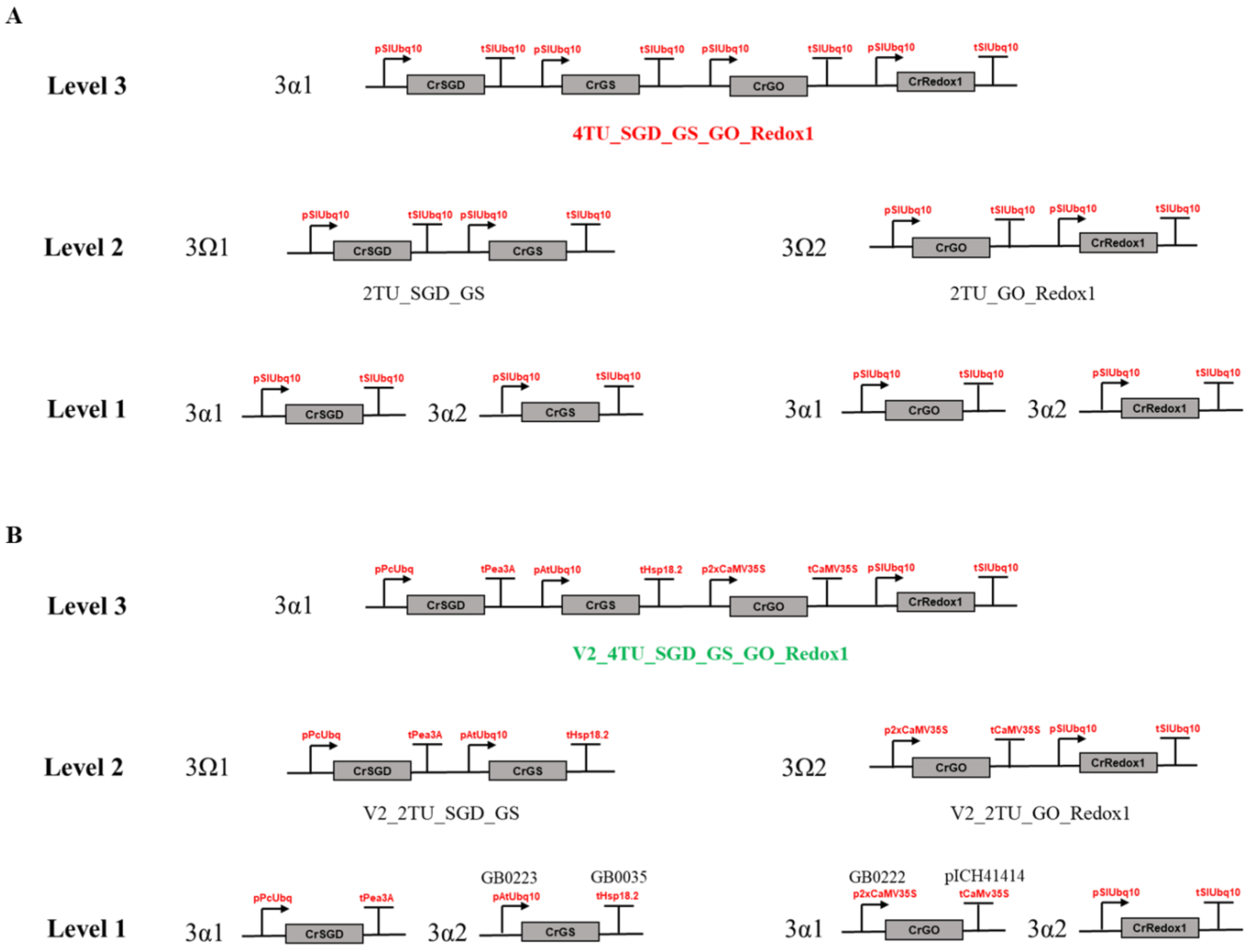

Supplementary Figure 9. Vectors used to build the 4 transcriptional unit assemblies. Level 0 vector containing standardized parts GBXXXX are available from the Golden Braid repository (https://gbcloning.upv.es/), pICH41414 comes from the MoClo Toolbox.[1] Level 0 vectors containing parts pSlUbq10[2], tSlUbq10, pPcUbq[3], tPea3A[4] were domesticated using the standard Golden Braid domestication protocol.[5] Vector backbones used (3α1/3α2; 3Ω1/3Ω2) are marked in each construct. **A.** Intermediate vectors used to construct 4TU_SGD_GS_GO_Redox1. **B.** Intermediate vectors used to construct V2_4TU_SGD_GS_GO_Redox1.

Supplementary Table 1. Sequences of *C. roseus* biosynthetic gene coding sequences and regulatory element sequences used in the study.

Gene	GenBank Accession	Domesticated coding sequence
SGD	AF 112 888	ATGGGTTCTAAGGATGATCAATCACTTGTCGTGGCTATCAGTCCTGCCGCCGAGCCTAACGGGAACCACTCA<br>GTTCCTATACCGTTTGCATATCCGAGCATACCTATACAGCCGCGAAAACATAATAAACCTATAGTACACAGG<br>CGGGACTTTCCTTCTGACTTTATTCTTGGAGCTGGCGGTTCCGCGTACCAATGCGAAGGCGCTTACAACGAGG<br>GAAATAGAGGACCAAGCATTTGGGACACATTTACTAATAGATACCCTGCAAAGATTGCAGACGGCAGTAACG<br>GAAACCAGGCAATTAACAGCTATAACTTATACAAAGAGGACATAAAGATAATGAAACAGACTGGGCTTGAG<br>AGCTACCGATTCTCTATCTCTTGGTCCCGAGTTCTGCCTGGTGGAAACTTGAGTGGCGGGGTTAACAAGGACG<br>GAGTGAAATTTTACCACGATTTCATTGACGAACTACTGGCAAACGGTATTAAGCCTTTCGCCACCTTGTTTCA<br>TTGGGACCTGCCTCAGGCGTTAGAAGATGAATACGGTGGATTTCTCAGCGACCGCATAGTTGAGGACTTCAC<br>AGAATACGCTGAGTTCTGTTTCTGGGAGTTTGGCGATAAGGTTAAGTTCTGGACAACATTTAACGAGCCTCAC<br>ACATACGTCGCTTCAGGGTACGCTACGGGAGAGTTCGCCCCCGGCAGGGGCGGAGCTGACGGAAAAGGTGA<br>GCCAGGAAAGGAGCCTTACATTGCTACCCACAACCTTTTGCTCTCACATAAGGCAGCAGTAGAGGTTTACAG<br>AAAGAACTTCCAAAAGTGCCAGGGCGGAGAGATCGGCATAGTCTTAAACTCTATGTGGATGGAACCCCTTAA<br>CGAGACTAAGGAAGATATCGACGCACGCGAGCGTGGACTCGACTTTATGCTTGGTTGGTTTATTGAACCTCTC<br>ACTACAGGAGAGTATCCTAAGAGTATGCGGGCACTCGTTGGTAGTCGGTTGCCTGAGTTCTCCACAGAGGTG<br>TCAGAGAAGCTCACTGGCTGTTACGACTTCATTGGTATGAACTACTACACTACAACATACGTAAGCAACGCC<br>GATAAGATCCCTGACACCCCTGGATATGAAACCGACGCAAGAATCAACAAGAACATATTCGTGAAGAAGGT<br>AGACGGTAAAGAGGTTAGAATAGGCGAGCCATGTTACGGTGGTTGGCAACACGTGGTGCCTTCCGGTCTATA<br>TAACCTACTTGTGTATACCAAAGAAAAGTATCACGTACCCGTTATATACGTTAGCGAGTGCGGCGTAGTAGA<br>AGAGAACAGGACAAATATTTTGTTGACTGAGGGCAAGACAAATATCCTTTTGACTGAGGCCAGACATGACAA<br>GCTTCGAGTGGACTTCTTGCAGTCACACCTAGCCTCAGTTCGGGACGCAATAGACGACGGAGTCAACGTTAA<br>GGGGTTCTTCGTATGGAGTTTCTTTGATAATTTTGAGTGGAACCTTGGTTACATCTGTAGATACGGTATAATTC<br>ACGTAGACTACAAGACATTCCAGCGATACCCTAAAGACTCCGCTATTTGGTATAAGAACTTTATCTCAGAGG<br>GCTTCGTGACTAACACCGCAAAGAAACGTTTTAGGGAAGAGGACAAGCTGGTAGAACTTGTTAAGAAACAG<br>AAGTATTAA
GS	MF 770 507	ATGGCCGGAGAAACAACCAAACTCGACCTTTCAGTGAAGGCTGTGGGATGGGGTGCTGCAGATGCATCTGGT<br>GTCCTTCAGCCCATCAAGTTCTATAGAAGAGTCCCTGGTGAACGGGATGTGAAGATTAGAGTTTTGTACTCTG<br>GTGTTTGCAATTTCGATATGGAAATGGTCAGAAACAAGTGGGGTTTCACCAGATATCCTTATGTGTTTGGACA<br>TGAAACTGCCGGTGAGGTGGTAGAAGTTGGCAGCAAAGTAGAGAAATTCAAGGTTGGAGACAAGGTAGCTG<br>TGGGATGTATGGTCGGATCTTGTGGTCAATGTTATAATTGTCAAAGTGGAATGGAGAATTACTGCCCAGAGC<br>CCAATATGGCTGATGGATCTGTTTACCGTGAGCAAGGGGAACGATCCTATGGGGGTTGTTCAAATGTGATGG<br>TTGTTGATGAAAAGTTCGTCCTCCGATGGCCCGAAAACTTGCCTCAAGATAAAGGGGTTGCTCTCCTTTGTGC<br>TGGGGTTGTTGTTTATAGCCCAATGAAACATTTGGGACTCGATAAGCCAGGAAAGCATATTGGGGTTTTCGG<br>GCTGGGAGGTCTTGGTTCTGTTGCTGTTAAGTTTATTAAGGCTTTTGGTGGTAAGGCTACTGTTATTAGTACAT<br>CAAGGCGTAAGGAGAAGGAAGCCATTGAAGAACATGGTGCTGATGCTTTTGTTGTCAACACTGACTCTGAGC<br>AATTGAAGGCTCTGGCAGGTACTATGGATGGTGTTGTGGACACCACCCCAGGTGGCCGCACTCCTATGTCACT<br>TATGCTCAATTTGCTCAAGTTTGACGGCGCGGTTATGCTCGTAGGTGCACCGGAGTCGCTATTTGAGCTCCCT<br>GCGGCACCTCTCATTATGGGAAGGAAAAAGATAATCGGAAGTTCCACTGGAGGCCTCAAAGAGTACCAAGA<br>AATGCTTGATTTCGCAGCCAAACATAACATTGTATGTGATACTGAAGTTATTGGGATTGACTATCTCAGCACT<br>GCTATGGAACGTATCAAGAATTTGGATGTCAAGTACCGTTTTGCGATTGACATTGGAAATACATTGAAATTTG<br>AGGAATAA

GO	MF770508	ATGGAGTTTTCTTTCTCCTCACCAGCTCTCTACATAGTTTATTTCTTGCTTTTCTTTGTGGTAAGGCAATTATTG<br>AAACCCAAAAGTAAGAAAAAAATTGCCACCAGGTCCAAGAACACTACCCTTAATTGGAAACCTTCATCAACTC<br>TCGGGACCTTTACCTCATCGTACCCTAAAAAATTTGTCCGATAAACATGGTCCTTTGATGCACGTGAAAATGG<br>GCGAACGTTCGGCAATTATAGTATCAGATGCAAGAATGGCAAAAATAGTTCTTCATAATAACGGTTTAGCCG<br>TTGCAGATCGGTCAGTAAATACTGTCGCAAGTATTATGACTTATAATAGTTTGGGTGTTACCTTTGCTCAGTA<br>TGGAGATTACTTAACAAAATTACGTCAAATCTATACTTTGGAACTTTTAAGTCAGAAAAAAGTTCGATCTTTC<br>TACAGTTGTTTTGAAGATGAACTCGATACTTTTGTTAAGTCAATTAAGTCTAACGTTGGACAACCTATGGTTTT<br>GTACGAGAAAGCTTCTGCTTATTTGTATGCTACCATTTGTAGAACTATTTTTGGGAGTGTTTGCAAGGAAAAG<br>GAGAAAATGATTAAGATAGTGAAGAAAACGTCGTTACTGTCTGGAACACCACTTAGATTGGAGGATCTTTTT<br>CCTTCTATGAGCATTTTTTGTAGATTTAGTAAAACTTTGAATCAATTGAGAGGACTTTTGCAAGAAATGGATG<br>ATATTTTGGAAGAAATCATAGTTGAAAGGGAAAAGGCTTCTGAAGTCTCAAAAGAGGCAAAGGATGATGAA<br>GATATGCTTAGTGTTCTGTTGAGGCATAAATGGTACAATCCAAGTGGTGCTAAATTCAGAATCACTAATGCAG<br>ACATCAAAGCCATTATCTTTGAGTTGATCTTAGCAGCAACCCTAAGTGTAGCAGATGTTACAGAATGGGCTAT<br>GGTTGAAATTCTAAGGGATCCCAAAAGTTTGAAAAAAGTGTACGAAGAAGTACGAGGTATTTGTAAAGAAA<br>AGAAAAGAGTGACAGGATACGACGTTGAAAAAATGGAATTTATGCGTCTTTGTGTGAAGGAATCAACAAGA<br>ATACATCCAGCTGCTCCTCTTTTAGTTCCTAGAGAATGCAGAGAAGATTTTGAAGTAGATGGATACACAGTTC<br>CAAAAGGGGCTTGGGTTATAACAAATTGCTGGGCTGTTCAAATGGATCCTACAGTATGGCCCGAACCAGAGA<br>AGTTTGATCCAGAAAGATATATTCGTAACCCAATGGATTTCTATGGAAGTAACTTTGAATTGATACCATTTGG<br>GACAGGTAGAAGAGGATGTCCTGGTATTTTATATGGAGTGACAAATGCTGAGTTTATGTTGGCTGCTATGTTT<br>TATCATTTTGATTGGGAAATAGCAGATGGTAAAAAACCAGAAGAAATTGATTTGACTGAGGACTTTGGTGCT<br>GGTTGCATTATGAAGTATCCTTTGAAATTGGTTCCTCATCTTGTTAACGATTAA
Redox1	MF770509	ATGGCTGATCGCGTGAAAACCGTAGGATGGGCAGCTCACGACAGCTCCGGCTTCCTCTCTCCCTTCCAATTCA<br>CTCGAAGGGCAACAGGTGAAGAAGATGTGAGGTTGAAGGTGTTGTACTGTGGTGTCTGTCACTCAGACCTTC<br>ATAACATCAAGAACGAAATGGGATTCACCTCCTACCCTTGTGTCCCCGGGCATGAGGTTGTGGGGGAAGTGA<br>CGGAAGTGGGGAACAAAGTAAAGAAATTCATAATTGGTGATAAAGTTGGGGTTGGATTATTCGTTGACTCAT<br>GTGGCGAATGCGAACAATGTGTGAATGATGTAGAAACCTATTGTCCCAAATTGAAAATGGCTTATTTATCCAT<br>TGATGATGATGGAACTGTGATTCAAGGAGGGTACTCAAAGGAAATGGTCATCAAAGAACGCTACGTTTTCCG<br>GTGGCCGGAAAATCTTCCTCTACCCGCCGGTACACCGCTTCTGGGTGCCGGTAGTACAGTTTATAGTCCAATG<br>AAATACTATGGACTTGATAAGTCAGGACAACATCTAGGAGTTGTTGGCCTTGGTGGACTTGGTCATTTAGCTG<br>TTAAATTTGCAAAGGCTTTTGGACTTAAAGTTACTGTCATTAGTACCTCTCCTAGCAAGAAGGATGAAGCCAT<br>CAATCACCTTGGTGCTGATGCATTTTTAGTTAGCACTGATCAAGAGCAAACCCAGAAAGCAATGAGCACAAT<br>GGATGGCATAATTGACACAGTATCAGCTCCTCATGCATTGATGCCATTGTTTTCCCTATTGAAACCAAATGGG<br>AAGCTAATTGTTGTTGGTGCACCAAATAAACCAGTTGAATTAGATATTCTATTTCTTGTCATGGGAAGGAAAA<br>TGCTCGGAACATCTGCTGTTGGGGGAGTGAAGGAGACACAAGAGATGATCGATTTTGCAGCAAAACATGGTA<br>TAGTTGCAGATGTAGAAGTTGTGGAAATGGAGAATGTGAACAATGCAATGGAGCGGCTTGCGAAGGGGGAT<br>GTTAGATATAGATTTGTTCTTGATATTGGGAATGCAACAGTAGCTGTCGGTTAA
Redox2	MF770510	ATGGAAAAGCAAGTTGAGATCCCTGAAGTAGAATTGAATTCAGGACATAAAATGCCAATTGTGGGATACGG<br>AACATGCGTGCCAGAACCCATGCCACCGTTGGAAGAACTAACCGCAATCTTCTTAGATGCAATAAAGGTTGG<br>TTACCGGCACTTCGACACGGCTTCGAGTTACGGCACAGAGGAGGCACTTGGTAAAGCCATAGCTGAGGCTAT<br>AAACAGCGGTTTGGTTAAAAGTAGGGAGGAATTCTTCATCAGTTGTAAGCTGTGGATTGAAGATGCAGATCA<br>TGACCTTATCTTGCCTGCCCTCAACCAGTCACTTCAGATTCTTGGGGTTGATTATTTGGATCTATATATGATAC<br>ACATGCCGGTGAGAGTGAGGAAAGGTGCTCCCATGTTCAATTATTCAAAAGAGGATTTCCTTCCATTTGACAT<br>ACAAGGTACATGGAAGGCCATGGAAGAGTGCAGCAAACAAGGATTGGCCAAGTCTATTGGTGTCAGCAACT<br>ACTCTGTTGAAAAACTCACTAAACTCCTAGAAACCTCCACTATTCCCCCCGCCGTTAATCAGGTTGAGATGAA<br>TGTAGCTTGGCAACAGAGAAAATTGCTGCCATTTTGCAAAGAGAAAAACATTCATATAACATCATGGTCCCC<br>ACTCCTATCTTATGGTGTCGCTTGGGGAAGTAATGCTGTCATGGAAAATCCTGTTCTCCAACAAATTGCGGCT<br>TCCAAAGGCAAAACTGTGGCACAGGTGGCACTAAGATGGATATACGAGCAAGGAGCAAGTCTCATTACAAG<br>GACTTCCAACAAGGACAGAATGTTTGAAAATGTTCAAATTTTTGATTGGGAACTCAGTAAAGAAGAATTGGA<br>TCAAATTCATGAAATCCCACAACGTAGGGGTACTTTAGGTGAAGAATTTATGCATCCAGAAGGACCGATCAA<br>ATCCCCAGAGGAGCTTTGGGATGGAGACTTGTAA
SAT	MF770511	ATGGCACCCCAGATGCAGATATTGTCAGAGGAACTGATTCAACCATCATCTCCGACACCCCAAACCTTGAAA<br>ACCCATAAACTTTCCCATCTTGATCAAGTTTTATTAACATGTCATATCCCTATTATTCTCTTTTATCCAAATCA<br>ATTGGACTCAAATCTCGATCGAGCCCAAAGATCTGAGAATCTAAAACGATCTTTATCAACAGTGTTAACTCA<br>ATTTTACCCTTTAGCCGGAAGAATCAATATAAATTCTTCCGTAGATTGTAATGATTCCGGAGTCCCTTTTCTTG<br>AAGCTCGAGTTCATTCCCAACTCTCAGAAGCAATTAAAAATGTCGCCATAGATGAACTCAATCAATACCTGC<br>CATTCCAACCTTATCCCGGTGGGGAGGAAAGTGGGTTGAAAAAAGATATCCCCTTAGCTGTAAAAATCAGTT<br>GTTTCGAATGTGGCGGAACAGCAATTGGGGTCTGTATTTCCCACAAGATTGCCGATGCGTTGTCCTTGGCTAC<br>CTTCCTCAATTCATGGACCGCAACATGCCAGGAAGAGACTGATATTGTTCAACCTAATTTCGATCTGGGATCC<br>CATCATTTTCCGCCTATGGAAAGCATCCCAGCACCTGAATTTCTACCGGATGAAAATATTGTGATGAAACGGT<br>TCGTGTTCGATAAAGAAAAATTAGAAGCTCTAAAAGCCCAATTAGCTTCCTCTGCAACAGAAGTGAAGAATT<br>CAAGTCGGGTACAAATTGTTATTGCTGTTATATGGAAGCAATTCATTGACGTGACCCGGGCAAAATTCGATAC<br>CAAGAACAAATTAGTAGCAGCTCAAGCAGTGAATTTGAGATCAAGAATGAATCCACCATTTCCTCAATCTGC<br>TATGGGGAATATAGCCACAATGGCATATGCCGTTGCAGAGGAGGATAAGGATTTTTCAGATCTTGTCGGTCC<br>ATTGAAAACCAGTCTTGCAAAAATTGATGATGAACATGTTAAAGAATTACAAAAAGGAGTAACATATTTGGA<br>TTATGAAGCTGAACCACAAGAATTGTTCTCTTTTAGTAGTTGGTGCAGGCTTGGGTTTTATGATTTGGATTTTG<br>GATGGGGAAAGCCTGTTTCTGTTTGTACAACAACTGTGCCTATGAAGAATTTGGTATATTTGATGGATACAAG<br>AAATGAAGATGGAATGGAAGCATGGATCAGCATGGCTGAAGATGAGATGTCTATGCTTTCCTCTGATTTTCTT<br>TCACTTCTGGACACTGATTTTAGCAATTGA

PAS	MH213134	ATGATAAAAAAAGTCCCAATAGTTCTTTCAATTTTCTGCTTTCTTCTTCTACTCTCATCATCCCATGGCTCAAT<br>TCCTGAAGCTTTTCTCAATTGTATTTCCAATAAATTTTCATTAGATGTATCCATTTTAAACATTCTTCATGTTCC<br>CAGCAATTCTTCCTATGATTCTGTTCTCAAATCTACTATCCAAAATCCAAGATTCCTCAAATCACCCAAGCCCT<br>TAGCTATAATCACCCCAGTACTTCACTCCCATGTCCAATCTGCTGTTATCTGTACCAAACAAGCCGGTTTACA<br>AATTAGAATCCGAAGCGGAGGAGCTGATTACGAGGGCTTATCCTATCGTTCTGAGGTTCCCTTTATTCTGCTA<br>GATCTCCAGAATCTTCGATCAATTTCCGTTGATATTGAGGACAACAGCGCTTGGGTCGAATCAGGAGCAACA<br>ATTGGTGAATTCTATCATGAGATAGCTCAGAACAGCCCTGTTCATGCGTTTCCAGCTGGAGTCTCTTCCTCTGT<br>TGGAATTGGCGGCCATTTGAGTAGCGGCGGTTTTGGTACATTGCTTCGGAAATATGGATTAGCAGCCGATAAT<br>ATAATCGATGCAAAAATTGTTGATGCCAGAGGCAGAATTCTTGATAGGGAATCAATGGGAGAAGATCTATTT<br>TGGGCTATTAGAGGAGGAGGAGGAGCTAGTTTTGGTGTTATAGTTTCTTGGAAGGTTAAACTTGTAAAAGTC<br>CCTCCGATGGTAACTGTTTTCATCTTGTCCAAGACTTATGAAGAAGGAGGTTTAGATCTTCTACACAAATGGC<br>AATATATAGAACACAAACTCCCTGAAGATTTATTCCTTGCTGTAAGCATCATGGATGATTCATCTAGTGGAAA<br>TAAAACACTTATGGCAGGTTTTATGTCTCTGTTTCTTGGAAAAACAGAGGACCTTCTGAAAGTAATGGCGGAA<br>AATTTCCCACAACTTGGATTGAAAAAGGAAGATTGCTTAGAAATGAATTGGATTGATGCAGCAATGTATTTTT<br>CAGGACACCCAATTGGAGAATCCCGATCTGTGCTTAAAAACCGAGAATCTCATCTTCCAAAGACATGCGTTT<br>CGATCAAATCAGACTTTATTCAAGAACCACAATCCATGGATGCATTGGAAAAGTTATGGAAGTTTTGTAGGG<br>AAGAAGAAAATAGTCCCATAATACTGATGCTTCCACTGGGGGGAATGATGAGTAAAATATCAGAATCAGAA<br>ATCCCATTTCCTTACAGAAAAGATGTGATTTACAGTATGATATACGAAATAGTTTGGAATTGTGAAGATGATG<br>AATCATCGGAAGAATATATCGATGGATTGGGAAGGCTTGAGGAATTAATGACTCCATATGTGAAACAACCAA<br>GAGGTTCTTGGTTCAGCACCAGAAACCTTTATACCGGTAAAAATAAAGGTCCAGGAACAACTTATTCCAAAG<br>CTAAAGAATGGGGATTTCGGTATTTTAATAATAATTTCAAAAAGTTGGCCCTTATCAAAGGACAAGTTGATCC<br>AGAAAACTTCTTCTACTATGAACAAAGCATTCCCCCTCTCCATTTACAAGTCGAACTTTGA
DPAS	KU865331	ATGAATTCCTCAACTGATCCAACTTCAGATGAGACTATTTGGGATCTTTCTCCATATATTAAAATTTTCAAAG<br>ATGGAAGAGTAGAAAGACTCCATAATAGTCCTTATGTTCCCCCATCACTTAATGATCCAGAAACTGGCGTTTC<br>TTGGAAAGATGTCCCGATTTCATCACAAGTTTCCGCTAGGGTATACATTCCAAAAATCAGCGACCATGAAAA<br>ACTCCCTATTTTTGTGTATGTGCATGGGGCTGGCTTTTGTCTAGAATCTGCCTTCAGATCATTTTTCCACACTTT<br>TGTCAAACACTTCGTAGCCGAAACCAAAGTTATTGGGGTTTCGATTGAATATAGACTTGCCCCAGAGCACCTT<br>TTACCCGCAGCTTATGAAGATTGTTGGGAAGCCCTTCAATGGGTTGCTTCTCATGTGGGTCTTGACAATTCCG<br>GCCTAAAGACAGCTATTGATAAAGATCCATGGATAATAAACTATGGTGATTTCGATAGACTGTATTTGGCGG<br>GTGACAGTCCTGGTGCTAATATTGTTCACAACACACTTATCAGAGCTGGAAAAGAGAAACTGAAGGGCGGAG<br>TGAAAATTTTGGGGGCAATTCTTTACTACCCATATTTCATTATCCCAACCAGCACGAAACTTAGTGATGATTT<br>TGAGTATAACTACACATGTTACTGGAAATTGGCTTATCCAAATGCTCCTGGCGGGATGAATAACCCAATGAT<br>AAACCCCATAGCTGAAAATGCTCCAGACTTGGCTGGATACGGTTGCTCGAGGTTGTTGGTTACCCTGGTTTCC<br>ATGATTTCAACGACTCCAGATGAGACTAAAGACATAAATGCGGTTTATATTGAGGCATTAGAAAAGAGTGGA<br>TGGAAAGGGGAATTGGAAGTGGCTGATTTTGACGCAGATTATTTTGAACTCTTCACCTTGGAAACGGAGATG<br>GGCAAGAATATGTTCAGACGTTTAGCATCTTTCATCAAACATGAGTAA
CS	MF770512	ATGAATTCCTCAACTGATCCAACTTCAGATGAGACTATTTGGGATCTTTCTCCATATATTAAAATTTTCAAAG<br>ATGGAAGAGTAGAAAGACTCCATAATAGTCCTTATGTTCCCCCATCACTTAATGATCCAGAAACTGGCGTTTC<br>TTGGAAAGATGTCCCGATTTCATCACAAGTTTCCGCTAGGGTATACATTCCAAAAATCAGCGACCATGAAAA<br>ACTCCCTATTTTTGTGTATGTGCATGGGGCTGGCTTTTGTCTAGAATCTGCCTTCAGATCATTTTTCCACACTTT<br>TGTCAAACACTTCGTAGCCGAAACCAAAGTTATTGGGGTTTCGATTGAATATAGACTTGCCCCAGAGCACCTT<br>TTACCCGCAGCTTATGAAGATTGTTGGGAAGCCCTTCAATGGGTTGCTTCTCATGTGGGTCTTGACAATTCCG<br>GCCTAAAGACAGCTATTGATAAAGATCCATGGATAATAAACTATGGTGATTTCGATAGACTGTATTTGGCGG<br>GTGACAGTCCTGGTGCTAATATTGTTCACAACACACTTATCAGAGCTGGAAAAGAGAAACTGAAGGGCGGAG<br>TGAAAATTTTGGGGGCAATTCTTTACTACCCATATTTCATTATCCCAACCAGCACGAAACTTAGTGATGATTT<br>TGAGTATAACTACACATGTTACTGGAAATTGGCTTATCCAAATGCTCCTGGCGGGATGAATAACCCAATGAT<br>AAACCCCATAGCTGAAAATGCTCCAGACTTGGCTGGATACGGTTGCTCGAGGTTGTTGGTTACCCTGGTTTCC<br>ATGATTTCAACGACTCCAGATGAGACTAAAGACATAAATGCGGTTTATATTGAGGCATTAGAAAAGAGTGGA<br>TGGAAAGGGGAATTGGAAGTGGCTGATTTTGACGCAGATTATTTTGAACTCTTCACCTTGGAAACGGAGATG<br>GGCAAGAATATGTTCAGACGTTTAGCATCTTTCATCAAACATGAGTAA
TS	MF770513	ATGGGTTCCTCAGATGAGACTATTTTTGATCTTCCTCCATACATCAAAGTATTCAAAGATGGAAGAGTAGAAA<br>GACTCCATTCTTCCCCATATGTTCCCCCATCTCTTAATGATCCAGAAACCGGTGGAGTCTCTTGGAAAGACGT<br>CCCAATTTCTTCAGTAGTTTCAGCTAGAATTTACCTTCCTAAAATCAACAACCATGATGAAAAACTCCCCATT<br>ATAGTCTATTTCCATGGAGCTGGTTTTTGTCTTGAATCGGCCTTCAAATCATTTTTCCACACTTATGTGAAACA<br>CTTTGTAGCAGAAGCCAAAGCTATTGCGGTTTCTGTTGAGTTCAGGCTCGCCCCTGAAAACCATTTACCCGCA<br>GCTTATGAAGATTGCTGGGAAGCCCTTCAATGGGTTGCTTCTCATGTGGGGCTCGACATTTCCAGCTTGAAAA<br>CTTGTATTGATAAAGATCCATGGATAATCAACTATGCCGATTTCGATAGACTCTATTTGTGGGGTGATAGCAC<br>CGGTGCCAATATTGTTCACAACACACTTATCAGATCTGGTAAAGAGAAATTGAACGGCGGCAAAGTGAAGAT<br>TTTGGGGGCAATTCTTTACTACCCATATTTCTTAATCAGGACGAGTTCAAAACAGAGTGATTATATGGAGAAT<br>GAGTATAGATCTTACTGGAAATTGGCTTACCCAGATGCTCCTGGTGGAAATGATAACCCAATGATAAACCCT<br>ACAGCTGAGAATGCTCCTGATCTGGCTGGATATGGTTGCTCGAGGTTGCTGATTTCCATGGTTGCCGATGAAG<br>CTAGAGATATAACTCTTCTTTATATTGATGCATTGGAAAAGAGTGGATGGAAAGGTGAATTAGATGTGGCTG<br>ATTTTGATAAACAGTATTTTGAACTGTTTGAAATGGAAACAGAGGTTGCCAAGAACATGCTCAGACGTTTAG<br>CTTCTTTCATCAAGTAA

pSlUbq10	-	GGAGGTCAACTACCCCAATTTAAATTTTATTTGATTAAGATATTTTTATGGACCTACTTTATAATTAAAAATAT<br>TTTCTATTTGAAAAGGAAGGACAAAAATCATACAATTTTGGTCCAACTACTCCTCTCTTTTTTTTTTTGGCTTT<br>ATAAAAAAGGAAAGTGATTAGTAATAAATAATTAAATAATGAAAAAAGGAGGAAATAAAATTTTCGAATTA<br>AAATGTAAAAGAGAAAAAGGAGAGGGAGTAATCATTGTTTAACTTTATCTAAAGTACCCCAATTCGATTTTA<br>CATGTATATCAAATTATACAAATATTTTATTAAAATATAGATATTGAATAATTTTATTATTCTTGAACATGTAA<br>ATAAAAATTATCTATTATTTCAATTTTTATATAAACTATTATTTGAAATCTCAATTATGATTTTTTAATATCACT<br>TTCTATCCATGATAATTTCAGCTTAAAAAGTTTTGTCAATAATTACATTAATTTTGTTGATGAGGATGACAAG<br>ATTTCGGTCATCAATTACATATACACAAATTGAAATAGTAAGCAACTTGATTTTTTTTCTCATAATGATAATG<br>ACAAAGACACGAAAAGACAATTCAATATTCACATTGATTTATTTTTATATGATAATAATTACAATAATAATAT<br>TCTTATAAAGAAAGAGATCAATTTTGACTGATCCAAAAATTTATTTATTTTTACTATACCAACGTCACTAATT<br>ATATCTAATAATGTAAAACAATTCAATCTTACTTAAATATTAATTTGAAATAAACTATTTTTATAACGAAATT<br>ACTAAATTTATCCAATAACAAAAAGGTCTTAAGAAGACATAAATTCTTTTTTTGTAATGCTCAAATAAATTTG<br>AGTAAAAAAGAATGAAATTGAGTGATTTTTTTTTAATCATAAGAAAATAAATAATTAATTTCAATATAATAA<br>AACAGTAATATAATTTCATAAATGGAATTCAATACTTACCTCTTAGATATAAAAAATAAATATAAAAATAAA<br>GTGTTTCTAATAAACCCGCAATTTAAATAAAATATTTAATATTTTCAATCAAATTTAAATAATTATATTAAAA<br>TATCGTAGAAAAAGAGCAATATATAATACAAGAAAGAAGATTTAAGTACAATTATCAACTATTATTATACTC<br>TAATTTTGTTATATTTAATTTCTTACGGTTAAGGTCATGTTCACGATAAACTCAAAATACGCTGTATGAGGAC<br>ATATTTTAAATTTTAACCAATAATAAAACTAAGTTATTTTTAGTATATTTTTTTGTTTAACGTGACTTAATTTTT<br>CTTTTCTAGAGGAGCGTGTAAGTGTCAACCTCATTCTCCTAATTTTCCCAACCACATAAAAAAAAAAATAAAGG<br>TAGCTTTTGCGTGTTGATTTGGTACACTACACGTCATTATTACACGTGTTTTCGTATGATTGGTTAATCCATGA<br>GGCGGTTTCCTCTAGAGTCGGCCATACCATCTATAAAATAAAGCTTTCTGCAGCTCATTTTTTCATCTTCTATC<br>TGATTTCTATTATAATTTCTCTGAATTGCCTTCAAATTTCTCTTTCAAGGTTAGAATTTTTCTCTATTTTTTGGT<br>TTTTGTTTGTTTAGATTCTGAGTTTAGTTAATCAGGTGCTGTTAAAGCCCTAAATTTTGAGTTTTTTTCGGTTGT<br>TTTGATGGAAAATACCTAACAATTGAGTTTTTTCATGTTGTTTTGTCGGAGAATGCCTACAATTGGAGTTCCTT<br>TCGTTGTTTTGATGAGAAAGCCCCTAATTTGAGTGTTTTTCCGTCGATTTGATTTTAAAGGTTTATATTCGAGT<br>TTTTTTCGTCGGTTTAATGAGAAGGCCTAAAATAGGAGTTTTTCTGGTTGATTTGACTAAAAAAGCCATGGAA<br>TTTTGTGTTTTTGATGTCGCTTTGGTTCTCAAGGCCTAAGATCTGAGTTTCTCCGGTTGTTTTGATGAAAAAGC<br>CCTAAAATTGGAGTTTTTATCTTGTGTTTTAGGTTGTTTTAATCCTTATAATTTGAGTTTTTTCGTTGTTCTGAT<br>TGTTGTTTTTATGAATTTTGCAG
tSlUbq10	-	GCTTGTTGTGGTTGTCTGGTTGCGTCTGTTGCCCGTTGTCTGTTGCCCATTGTGGTGGTTGTGTTTGTATGATG<br>GTCGTTAAGGATCATCAATGTGTTTTCGCTTTTTGTTCCATTCTGTTTCTCATTTGTGAATAATAATGGTATCTT<br>TATGAATATGCAGTTTGTGGTTTCTTTTCTGATTGCAGTTCTGAGCATTTTGTTTTTGCTTCCGTTTACTATACC<br>ACTTACAGTTTGCACTAATTTAGTTGATATGCGAGCCATCTGATGTTTGATGATTCAAATGGCGTTTATGTAA<br>CTCGTACCCGAGTGGATGGAGAAGAGCTCCATTGCCGGTTTGTTTCATGGGTGGCGGAGGGCAACTCCTGGG<br>AAGGAACAAAAGAAAAACCGTGATACGAGTTCATGGGTGAGAGCTCCAGCTTGATCCCTTCTCTGTCGATCA<br>AATTTGAATTTTTGGATCACGGCAGGCTCACAAGATAATCCAAAGTAAAACATAATGAATAGTACTTCTCAA<br>TGATCACTTATTTTTAGCAAATCAGCAATTGTGCATGTCAAATGATTTCGGTGTAAGAGAAAGAGTTGATGAA<br>TCAAAATATCTGTAGCTGGATCAAGAATCTGAGGCAGTTGTATGTATCAATGATCTTTCCGCTACAATGATGT<br>TAGCTATCCGAGTCAAATTGTTGTAGAATTGCATACTTCGGCATCACATTCTGGATGACATAATAAATAGGAA<br>GTCTTCAGATCCCTAAAAAATTGAGAGCTAATAACATTAGTCCTAGATGTAACTGGGTGACAACCAAGAAAG<br>AGACATGCAAATACTACTTTTGTTTGAAGGAGCATCCCTGGTTTGACATATTTTTTCTGAATATCAAACTTTGA<br>AACTCTACCTAGTCTAATGTCTAACGACAGATCTTACTGGTTTAACTGCAGTGATATCTACTATCTTTTGGAAT<br>GTTTTCTCCTTCAGTTATACATCAAGTTCCAAGATGCAGGTGTGCTTGATTGATGTACATGGCTGTGAGAAGT<br>GCATCCTGATGTTCAGATGATGGTTCATTCTAATGTCTTTTCCTTCAATCAGTTTTCTCAGTCTGACTTAGCTT<br>GTTTCATCTGCATGTTTGAATGTTCGTTTACTCATAGTAATTGCATTTTTGTAGCAGAACATATCATTGGTCAT<br>GGTTTCAACTGTGCGCGAGTCTTATGCTTATTCAAACTAGGAAAGCCTCCGTCTAGAGGGTACACGAGTTGTT<br>GCTCTGTGTGCGTCAGTCCATAGTATTAATCTTGCTAGTTGTAGTATATTGTTTATGTGGACTCGGAATTCATC<br>ATATGCTCCTTCTTTGCATCAAGTAAGGCAAGGTAATGTATAGAAGCTTTTTAACTCTTTCATGGAAGCTGGC<br>CTTTGCCAGCATACCATCCAGAAGATATCAACCCTGCATCTTGGCTGCCG
pPcUbq10	-	AAAAATTACGGATATGAATATAGGCATATCCGTATCCGAATTATCCGTTTGACAGCTAGCAACGATTGTACA<br>ATTGCTTCTTTAAAAAAGGAAGAAAGAAAGAAAGAAAAGAATCAACATCAGCGTTAACAAACGGCCCCGTT<br>ACGGCCCAAACGGTCATATAGAGTAACGGCGTTAAGCGTTGAAAGACTCCTATCGAAATACGTAACCGCAAA<br>CGTGTCATAGTCAGATCCCCTCTTCCTTCACCGCCTCAAACACAAAAATAATCTTCTACAGCCTATATATACA<br>ACCCCCCCTTCTATCTCTCCTTTCTCACAATTCATCATCTTTCTTTCTCTACCCCCAATTTTAAGAAATCCTCTC<br>TTCTCCTCTTCATTTTCAAGGTAAATCTCTCTCTCTCTCTCTCTCTCTGTTATTCCTTGTTTTAATTAGGTATGTA<br>TTATTGCTAGTTTGTTAATCTGCTTATCTTATGTATGCCTTATGTGAATATCTTTATCTTGTTCATCTCATCCGT<br>TTAGAAGCTATAAATTTGTTGATTTGACTGTGTATCTACACGTGGTTATGTTTATATCTAATCAGATATGAATT<br>TCTTCATATTGTTGCGTTTGTGTGTACCAATCCGAAATCGTTGATTTTTTTCATTTAATCGTGTAGCTAATTGTA<br>CGTATACATATGGATCTACGTATCAATTGTTCATCTGTTTGTGTTTGTATGTATACAGATCTGAAAACATCACT<br>TCTCTCATCTGATTGTGTTGTTACATACATAGATATAGATCTGTTATATCATTTTTTTTATTAATTGTGTATATA<br>TATATGTGCATAGATCTGGATTACATGATTGTGATTATTTACATGATTTTGTTATTTACGTATGTATATATGTA<br>GATCTGGACTTTTTGGAGTTGTTGACTTGATTGTATTTGTGTGTGTATATGTGTGTTCTGATCTTGATATGTTAT<br>GTATGTGCAGC

tPea3A	-	CAGGCCTCCCAGCTTTCGTCCGTATCATCGGTTTCGACAACGTTCGTCAAGTTCAATGCATCAGTTTCATTGCC<br>CACACACCAGAATCCTACTAAGTTTGAGTATTATGGCATTGGAAAAGCTGTTTTCTTCTATCATTTGTTCTGCT<br>TGTAATTTACTGTGTTCTTTCAGTTTTTGTTTTCGGACATCAAAATGCAAATGGATGGATAAGAGTTAATAAA<br>TGATATGGTCCTTTTGTTCATTCTCAAATTATTATTATCTGTTGTTTTTACTTTAATGGGTTGAATTTAAGTAAG<br>AAAGGAACTAACAGTGTGATATTAAGGTGCAATGTTAGACATATAAAACAGTCTTTCACCTCTCTTTGGTTAT<br>GTCTTGAATTGGTTTGTTTCTTCACTTATCTGTGTAATCAAGTTTACTATGAGTCTATGATCAAGTAATTATGC<br>AATCAAGTTAAGTACAGTATAGGCTT
pAtUbq10	-	GTCGACGAGTCAGTAATAAACGGCGTCAAAGTGGTTGCAGCCGGCACACACGAGTCGTGTTTATCAACTCAA<br>AGCACAAATACTTTTCCTCAACCTAAAAATAAGGCAATTAGCCAAAAACAACTTTGCGTGTAAACAACGCTC<br>AATACACGTGTCATTTTATTATTAGCTATTGCTTCACCGCCTTAGCTTTCTCGTGACCTAGTCGTCCTCGTCTTT<br>TCTTCTTCTTCTTCTATAAAACAATACCCAAAGAGCTCTTCTTCTTCACAATTCAGATTTCAATTTCTCAAAAT<br>CTTAAAAACTTTCTCTCAATTCTCTCTACCGTGATCAAGGTAAATTTCTGTGTTCCTTATTCTCTCAAAATCTTC<br>GATTTTGTTTTCGTTCGATCCCAATTTCGTATATGTTCTTTGGTTTAGATTCTGTTAATCTTAGATCGAAGACG<br>ATTTTCTGGGTTTGATCGTTAGATATCATCTTAATTCTCGATTAGGGTTTCATAGATATCATCCGATTTGTTCA<br>AATAATTTGAGTTTTGTCGAATAATTACTCTTCGATTTGTGATTTCTATCTAGATCTGGTGTTAGTTTCTAGTTT<br>GTGCGATCGAATTTGTCGATTAATCTGAGTTTTTCTGATTAACAG
tHsp18.2	-	ATATGAAGATGAAGATGAAATATTTGGTGTGTCAAATAAAAAGCTTGTGTGCTTAAGTTTGTGTTTTTTTCTT<br>GGCTTGTTGTGTTATGAATTTGTGGCTTTTTCTAATATTAAATGAATGTAAGATCTCATTATAATGAATAAACA<br>AATGTTTCTATAATCCATTGTGAATGTTTTGTTGGATCTCTTCTGCAGCATATAACTACTGTATGTGCTATGGT<br>ATGGACTATGGAATATGATTAAAGATAAG
p2xCaMV35S	-	ACTAGAGCCAAGCTGATCTCCTTTGCCCCGGAGATCACCATGGACGACTTTCTCTATCTCTACGATCTAGGAA<br>GAAAGTTCGACGGAGAAGGTGACGATACCATGTTCACCACCGATAATGAGAAGATTAGCCTCTTCAATTTCA<br>GAAAGAATGCTGACCCACAGATGGTTAGAGAGGCCTACGCGGCAGGTCTGATCAAGACGATCTACCCGAGT<br>AATAATCTCCAGGAGATCAAATACCTTCCCAAGAAGGTTAAAGATGCAGTCAAAAGATTCAGGACTAACTGC<br>ATCAAGAACACAGAGAAAGATATATTTCTCAAGATCAGAAGTACTATTCCAGTATGGACGATTCAAGGCTTG<br>CTTCATAAACCAAGGCAAGTAATAGAGATTGGAGTCTCTAAGAAAGTAGTTCCTACTGAATCAAAGGCCATG<br>GAGTCAAAAATTCAGATCGAGGATCTAACAGAACTCGCCGTGAAGACTGGCGAACAGTTCATACAGAGTCTT<br>TTACGACTCAATGACAAGAAGAAAATCTTCGTCAACATGGTGGAGCACGACACTCTCGTCTACTCCAAGAAT<br>ATCAAAGATACAGTCTCAGAAGACCAAAGGGCTATTGAGACTTTTCAACAAAGGGTAATATCGGGAAACCTC<br>CTCGGATTCCATTGCCCAGCTATCTGTCACTTCATCAAAAGGACAGTAGAAAAGGAAGGTGGCACCTACAAA<br>TGCCATCATTGCGATAAAGGAAAGGCTATCGTTCAAGATGCCTCTGCCGACAGTGGTCCCAAAGATGGACCC<br>CCACCCACGAGGAGCATCGTGGAAAAAGAAGACGTTCCAACCACGTCTTCAAAGCAAGTGGATTGATGTGAT<br>ATCTCCACTGACGTAAGGGATGACGCACAATCCCACTATCCTTCGCAATGAGACTTTTCAACAAAGGGTAAT<br>ATCGGGAAACCTCCTCGGATTCCATTGCCCAGCTATCTGTCACTTCATCAAAAGGACAGTAGAAAAGGAAGG<br>TGGCACCTACAAATGCCATCATTGCGATAAAGGAAAGGCTATCGTTCAAGATGCCTCTGCCGACAGTGGTCC<br>CAAAGATGGACCCCCACCCACGAGGAGCATCGTGGAAAAAGAAGACGTTCCAACCACGTCTTCAAAGCAAG<br>TGGATTGATGTGATATCTCCACTGACGTAAGGGATGACGCACAATCCCACTATCCTTCGCAAGACCCTTCCTC<br>TATATAAGGAAGTTCATTTCATTTGGAGAGGACTCCGGTATTTTTACAACAATTACCACAACAAAACAAACA<br>ACAAACAACATTACAATTTACTATTCTAGTCGA
tCaMV35S	-	CTCTAGCTAGAGTCGATCGACAAGCTCGAGTTTCTCCATAATAATGTGTGAGTAGTTCCCAGATAAGGGAATT<br>AGGGTTCCTATAGGGTTTCGCTCATGTGTTGAGCATATAAGAAACCCTTAGTATGTATTTGTATTTGTAAAAT<br>ACTTCTATCAATAAAATTTCTAATTCCTAAAACCAAAATCCAGTACTAAAATCCAGAT
pAtuNos	-	GAACCGCAACGTTGAAGGAGCCACTGAGCCGCGGGTTTCTGGAGTTTAATGAGCTAAGCACATACGTCAGAA<br>ACCATTATTGCGCGTTCAAAAGTCGCCTAAGGTCACTATCAGCTAGCAAATATTTCTTGTCAAAAATGCTCCA<br>CTGACGTTCCATAAATTCCCCTCGGTATCCAATTA

tAtuNos	-	GCTTGGAATGGATCTTCGATCCCGATCGTTCAAACATTTGGCAATAAAGTTTCTTAAGATTGAATCCTGTTGC<br>CGGTCTTGCGACGATTATCATATAATTTCTGTTGAATTACGTTAAGCATGTAATAATTAACATGTAATGCATG<br>ACGTTATTTATGAGATGGGTTTTTATGATTAGAGTCCCGCAATTATACATTTAATACGCGATAGAAAACAAAA<br>TATAGCGCGCAAACTAGGATAAATTATCGCGCDCGGTGTCATCTATGTTACTAGATCGGGAATTGCCAAGCT<br>AATTCTTGAAGACGAAAGGGCCTCGTGATACGCCTATTTTTATAGGTTAATGTCATGATAATAATGGTTTCTT<br>AGACGTCAGGTGGCACTTTTCGGGGAAATGTGCGCGGAACCCCTATTTGTTTATTTTTCTAAATACATTCAAA<br>TATGTATCCGCTCATGAGACAATAACCCTGATAAATGCTTCAATAATGGGACCGACTCG

Supplementary Table 2. The list of primers used in the study.

Primer ID	Sequence	Target
Q_001	AGAACGCTACGTTTTCCGGT	*C. roseus* Redox1 qPCR
Q_002	AAGTCCACCAAGGCCAACAA	*C. roseus* Redox1 qPCR
Q_003	GGTGTCGCTTGGGGAAGTAA	*C. roseus* Redox2 qPCR
Q_004	TGAGACTTGCTCCTTGCTCG	*C. roseus* Redox2 qPCR
Q_005	CAAGATTGCCGATGCGTTGT	*C. roseus* SAT qPCR
Q_006	GGATGCTTTCCATAGGCGGA	*C. roseus* SAT qPCR
Q_007	ACCGTTTGCATATCCGAGCA	*C. roseus* SGD qPCR
Q_008	TAAGCGCCTTCGCATTGGTA	*C. roseus* SGD qPCR
Q_011	TGATGCACGTGAAAATGGGC	*C. roseus* GO qPCR
Q_012	ACCGATCTGCAACGGCTAAA	*C. roseus* GO qPCR
Q_013	GATTGGCGGTATTGGTACTGTC	*N. benthamiana* EFα qPCR
Q_014	AGCTTCGTGGTGCATCTC	*N. benthamiana* EFα qPCR
Q_019	ATGGCCGGAGAAACAACCAA	*C. roseus* GS qPCR
Q_020	CCGTTCACCAGGGACTCTTC	*C. roseus* GS qPCR
0440	GCAGTAACGGAAACCAGGCA	*C. roseus* SGD sequencing
0441	GCCGCACTCGCTAACGTATA	*C. roseus* SGD sequencing
0442	GAAGAGTCCCTGGTGAACGG	*C. roseus* GS sequencing
0443	GCAGTGCTGAGATAGTCAATCC	*C. roseus* GS sequencing
0444	TTGCCACCAGGTCCAAGAAC	*C. roseus* GO sequencing
0445	TCTACCTGTCCCAAATGGTATCA	*C. roseus* GO sequencing
0446	GGCTTCCTCTCTCCCTTCCA	*C. roseus* Redox1 sequencing
0447	ACCATGTTTTGCTGCAAAATCG	*C. roseus* Redox1 sequencing
0448	TGCAGATATTGTCAGAGGAACTG	*C. roseus* SAT sequencing
0449	ACCAAATTCTTCATAGGCACAGT	*C. roseus* SAT sequencing
0450	TTGAATTCAGGACATAAAATGCCA	*C. roseus* Redox2 sequencing
0451	TGATCGGTCCTTCTGGATGC	*C. roseus* Redox2 sequencing
0452	TCTGCTTTCTTCTTCTACTCTCATCA	*C. roseus* PAS sequencing
0453	AGTAGAAGAAGTTTTCTGGATCAAC T	*C. roseus* PAS sequencing
0454	ATGGCCGGAAAATCAGCAGA	*C. roseus* DPAS sequencing
0455	CGCCCTTGTCTAAACGTTCC	*C. roseus* DPAS sequencing
0386	TGGGTTGCTTCTCATGTGGG	*C. roseus* CS sequencing
0387	TCACTCCGCCCTTCAGTTTC	*C. roseus* CS sequencing
0388	AGATTGCTGGGAAGCCCTTC	*C. roseus* TS sequencing
0389	TTGCCGCCGTTCAATTTCTC	*C. roseus* TS sequencing

Supplementary Table 3. The parameters used for detection of angryline, catharanthine and tabersonine using the EVOQ TQS.

**Compound name**	**Retention time [min]**	**Precursor ion *m/z***	**Fragment ions**	**Collision energy [eV]**
Angryline	4.30	337.19	168.00<br>228.00<br>196.00	34.00<br>18.00<br>25.00
Catharanthine	4.30	337.23	144.00<br>93.10<br>165.00<br>173.00<br>91.10	19.00<br>23.00<br>17.00<br>13.00<br>32.00
Tabersonine	4.00	337.19	168.00<br>227.90<br>305.00<br>196.00<br>169.90	35.00<br>19.00<br>19.00<br>26.00<br>31.00

Materials and methods

Media, chemicals and solvents used in the study

All HPLC solvents used in the study were purchased from Fisher Scientific and were HPLC-grade, apart from MeOH, which was LCMS-grade. Secologanin (ref. no. 50741), tryptamine (ref. no. 193747) and harmaline (ref. no. 51330) were purchased from Sigma-Aldrich. LB (Lennox broth) used for bacteria culturing was purchased from Formedium. Rifampicin, carbenicillin, gentamicin, chloramphenicol and spectinomycin antibiotics were purchased from Sigma-Aldrich.

Vector construction

All the multi transcriptional unit vectors were first assembled *in silico* using the Geneious Prime (V2022.1.1) and SnapGene (V6.0) software. The accession numbers of the *C. roseus* coding sequences (CDS) are listed in **Supplementary Table 1**.

Synthetic, domesticated coding sequences of SGD, GS, Redox1, Redox2 and SAT with suitable cloning adapter sequences were obtained from Twist Bioscience cloned into the pTwist chloroamphenicol high copy vector. The construct backbones and level 0 standardised parts used in the assemblies came from the Golden Braid (parts labelled as GBXXXX) and MoClo parts kits or were domesticated using the standard Golden Braid domestication procedure.[1,5,6] The intermediate vectors and all the parts used are shown in **Supplementary Figure 9**.

The level 0 vectors containing the biosynthetic gene coding sequences, the level 0 vectors containing standardised parts and the backbone vectors were used in Golden Gate assembly reactions. BsaI (New England Bioscience) was used for level 1 and level 3 construct assembly or Esp3I for level 2 construct assembly (ThermoFisher Scientific) together with T4 ligase (New England Bioscience) in T4 ligase buffer. The assembly was carried out by incubating the reaction mix at 37℃ for 5 min, followed by 5 min at 16℃ for 50 cycles. The reaction was then stopped by incubating at 65℃ for 10 min.

The single transcriptional unit constructs used in co-infiltration experiments, were build as follows; the domesticated coding sequences of the respective biosynthetic genes in pTwist chloroamphenicol plasmids were mixed with the pSlUbq10/tSlUbq10- containing vectors and 3α1/ 3α2 plasmids in the previously described standard Golden Gate reactions.

The single transcriptional unit constructs used in co-infiltration experiments, where expression was controlled by the AtuNos promoter and terminator pair were assembled as follows; the domesticated coding sequences of the respective biosynthetic genes in pTwist chloroamphenicol plasmids were mixed with the pAtuNos/tAtuNos level 0 part vectors (GB0072, GB0037) and 3α1 plasmids in the previously described standard Golden Gate reactions.

The resulting assembly mixes were used to directly transform chemically-competent *E. coli* Top10 cells. The transformants were plated on LB Lennox agar plates supplemented with X-gal and IPTG and a suitable antibiotic (spectinomycin 50 μg mL^{-1} or kanamycin 50 μg mL^{-1}). The plate was then incubated at 37 ℃ overnight and 4-6 white colonies were selected to inoculate 10 mL LB Lennox cultures supplemented with a suitable antibiotic. The cultures were incubated overnight at 37 ℃ with shaking. The bacterial pellet was then harvested using centrifugation and the recombinant plasmid was purified using the Wizard Miniprep kit (Promega), according to the manufacturer's instructions. The purified plasmid was subjected to a diagnostic restriction enzyme digest to confirm the successful assembly. To confirm the more complex assemblies, the plasmids were sequences using Sanger sequencing, service provided by Azenta, using the primers listed in **Supplementary Table 2**.

Synthesis of strictosidine

Strictosidine was synthesised from tryptamine and secologanin as previously described.[7] After completion, the reaction mixture was passed through a 6 mL Discovery DSC-C18 solid-phase extraction column (Supelco) and concentrated using a rotary evaporator. The mixture was then re-suspended in 100 % LCMS-grade MeOH and subjected to preparative HPLC purification on an Agilent 1260 Infinity II system. The MeOH solution was injected into a Phenomenex Luna column (5 μm, C18, 100 Å) in 500 μL aliquots. A gradient of 10 to 50% acetonitrile was applied over 28 min, followed by a 2 min 100% acetonitrile flow and 5 min re-equilibration to 10% acetonitrile and 90% of water 0.1% formic acid aqueous solution. The absorbance at 241 and 290 nm was monitored. The strictosidine fraction was then concentrated on a rotary evaporator and subjected to UPHLC-MS analysis to verify the purity of the extracted compound.

Agrobacterium tumefaciens transformation and culturing

Electrocompetent *A. tumefaciens* cells were transformed with up to 500 ng of the recombinant plasmids. Briefly, an aliquot of 50 μL of *A. tumefaciens* electrocompetent cells was mixed with the appropriate volume of the purified recombinant plasmid. The cells were transferred into an electroporation cuvette (0.2 cm gap) and subjected to an electrical pulse, using a micropulser (Bio-Rad). The cells were then re-suspended in 500 μL of LB Lennox medium and incubated at 28 ℃ with gentle shaking for 3-4 hours. The transformed cells were then plated on LB Lennox agar plates supplemented with rifampicin (100 μg mL^{-1}), gentamycin (10 μg mL^{-1}) and the vector selection antibiotic (either spectinomycin 50 μg mL^{-1} or kanamycin 50 μg mL^{-1}). The plates were incubated at 28 ℃ for 48 h. To confirm the successful transformation, 4-6 colonies of were selected and used for colony PCR. Briefly, colonies were picked with a toothpick, which was then dipped in 100 μL of molecular biology-grade water and incubated at 98 ℃ for 10 minutes. The resulting suspension was briefly centrifuged and used as a template for the PCR reactions. The Phire polymerase Master Mix (ThermoFisher Scientific) was used in the reactions with the same gene-specific primers that were used for sequencing (**Supplementary Table 2**).

The confirmed *A. tumefaciens* transformant colonies were used to inoculate 1 mL LB Lennox starter cultures supplemented with rifampicin, gentamycin (50 µg mL^{-1} and 25 µg mL^{-1} respectively) and the suitable vector-selection antibiotic and incubated at 28 ℃ overnight with shaking. The starter cultures were then diluted 1:100 in LB Lennox medium (same formulation as in the previous step) and incubated at 28 ℃ for 12 h. The bacteria were harvested by centrifugation and re-suspended in the infiltration buffer (50 mM MES pH 5.6, 10 mM $MgCl_2$, 200 µM acetosyringione, 50 mg mL^{-1} D-glucose). The strains were then brought to OD600 of 1 and mixed in appropriate combinations. The OD600 of the final inoculum was adjusted to 0.6 with the infiltration buffer and incubated in darkness, at room temperature for 2-3 hours prior to agroinfiltration.

Nicotiana benthamiana culturing conditions, agroinfiltration and leaf harvesting

N. benthamiana plants were grown in a low-nutrient F1 compost in a greenhouse, under long-day photoperiod conditions (16 h light, 8 h dark), at 22-25 ℃, 40-65% relative humidity for 3 weeks. The plants were then transferred to a controlled environment chamber (York) operating under the same conditions with light intensity set to 130 – 150 µE m^{-2} s^{-1}. The plants used for transient expression assays were between 3 and 4 weeks old, at the 2 fully expanded leaf pairs stage. The abaxial side of the second pair of leaves (counting from the apical meristem) of the plants was infiltrated with the *A. tumefaciens* inoculum using a needleless 1 mL syringe. The plants were then incubated in the growth chamber for 48 hours, prior to the strictosidine substrate infiltration. Aqueous strictosidine (200 μM) was infiltrated into the previously agro-infiltrated leaves. Around 1 mL of the substrate solution was infiltrated into each leaf. The substrate-infiltrated area was marked. The marked parts of the assay leaves were then harvested 48 h post substrate infiltration, snap-frozen in liquid N_2, ground to fine powder with a micropestle and stored at -80℃.

Extraction of metabolites from the harvested *N. benthamiana* leaves

The metabolites were extracted from the collected leaf tissue using LCMS-grade MeOH substituted with 1 μM of harmaline (Sigma Aldrich) as an internal standard. Aliquots of 200 μL of the extraction solution were added to each 100 mg of ground tissue. The mixture was then vortexed thoroughly, sonicated in a sonic bath for 15 min and spun down at 20 000 g for 20 min. The resulting extracts were subjected to UHPLC-MS analysis.

UHPLC-MS analysis of the *N. benthamiana* leaf extracts

The MeOH extracts were filtered through 0.3 μm PTFE filters and transferred into LCMS vials. The extracts were then analysed on a Bruker Impact II Q-TOF mass spectrometer connected to a Thermo UltiMate 3000 UHPLC system. The samples were passed through a Phenomenex Kinetex column (2.6 μm, XB-C18, 100 Å) at the column oven temperature of 40℃. A gradient of 10 to 30% of acetonitrile was applied over 7 min, followed by 1 min at 100% acetonitrile and 2.5 min

re-equilibration to 10% acetonitrile and 90% 0.1% formic acid aqueous solution. The flow rate used was 0.6 mL min $^{-1}$, the injection volume was 2 μL.

Ionisation was obtained using pneumatic-assisted electrospray ionisation in positive mode (ESI+) with 3500V capillary voltage, an end plate offset of 500 V, nebuliser pressure of 2.5 bar, drying nitrogen gas flow at temperature of 250℃ with 11 L min^{-1}. The data in the mass range of 80-1000 *m/z* were recorded at 12 Hz frequency with data-dependent MS/MS measurements. The stepping option model from 20 to 50 eV was used for collision energy application. Sodium formate solution in isopropanol was used as a calibrant at the beginning and the end of each run. The first 1.5 min of UHPLC gradient was directed into the waste, after which the sample was directed into the mass spectrometer.

The resulting data was analysed using Metaboscape (Bruker, V), MZmine (V2.52) and SiRIUS (V4.9.9) to identify and quantify the peak areas corresponding to PCA. [8,9] The levels of precondylocrapine acetate were determined based on a standard curve obtained by serially diluting the PAS reactions.

The extracts resulting from catharanthine and tabersonine production assays were analysed on the Bruker EVOQ TQS instrument connected to a Thermo UltiMate 3000 UHPLC system. The samples were passed through a Phenomenex Kinetex column (2.6 μm, XB-C18, 100 Å) at the column oven temperature of 40℃. A gradient of 10 to 30% of acetonitrile was applied over 7 min, followed by 1 min at 100% acetonitrile and 2.5 min re-equilibration to 10% acetonitrile and 90% 0.1% formic acid aqueous solution. The flow rate used was 0.6 mL min $^{-1}$, the injection volume was 2 μL. Ionisation was obtained using heated electrospray ionisation (HESI) in positive mode with 3500V capillary voltage and 450℃ heated probe temperature and 45 L min^{-1} probe gas flow and 50 L min^{-1} nebulizer gas flow. The cone temperature was kept at 350℃. Cone gas flow was set to 20 L min^{-1}. The ion transitions monitored for the detection of tabersonine, catharanthine and angryline and the corresponding collision energy used are listen in **Supplementary Table 3**.

PAS assay for precodylocarpine acetate standard curve construction

The PAS substrate stemmadenine acetate was prepared as previously described, from stemmadenine isolated by Professor Ivo J Curcino Vieira as reported previously.[10]

PAS was purified from *N. benthamiana* tissue as previously described.[10] The purified enzyme was used in assays as follows: 20 μM of enzyme was mixed with 200 μM FAD and 20 μM purified stemmmadenine acetate in a 50 mM HEPES pH 7.5 buffer of 100 μL total volume. The reaction was incubated at for 20 min at 30 ℃ with 650 rpm shaking in a thermomixer (Eppendorf). The reaction was then quenched with 1x volume of 100% MeOH, centrifuged at 25 000 G for 20 min and diluted serially by a factor of 2, to constitute the concentrations ranging from 10 to 1.625 μM of the precondylocarpine acetate product. The product was stored at - 20℃ until it was directly subjected to UHPLC-MS analysis.

RNA purification, cDNA synthesis and qPCR analysis of the harvested samples

Total RNA was isolated from ca. 50 mg of the harvested *N. benthamiana* tissue using the RNAeasy RNA-purification kit (Qiagen), following the manufacturer's instructions. The RNA was then subjected to DNAse digest (Sigma) and served as a template for cDNA synthesis, carried out using the High Capacity cDNA synthesis kit according to the manufacturer's instructions. The cDNA was then appropriately diluted and used as a template in the qPCR reactions using the FastSYBR master mix (Applied Biosystems) in a QuantStudio 1 cycler (Applied Biosystems). The primers used are listed in **Supplementary Table 2**. The results were then analysed in the QuantStudio software (Applied Biosystems, V1.5.2), using the $2^{-\Delta\Delta Ct}$ method.[11] The statistical analysis was done using SPSS software (IBM, V25).

Supplementary Materials and Methods References

(1) Engler, C., Youles, M., Gruetzner, R., Ehnert, T.-M., Werner, S., Jones, J. D. G., Patron, N. J., and Marillonnet, S. (2014) A Golden Gate modular cloning toolbox for plants. *ACS Synth. Biol. 3*, 839–843.

(2) Cárdenas, P. D., Sonawane, P. D., Heinig, U., Jozwiak, A., Panda, S., Abebie, B., Kazachkova, Y., Pliner, M., Unger, T., Wolf, D., Ofner, I., Vilaprinyo, E., Meir, S., Davydov, O., Gal-on, A., Burdman, S., Giri, A., Zamir, D., Scherf, T., Szymanski, J., Rogachev, I., and Aharoni, A. (2019) Pathways to defense metabolites and evading fruit bitterness in genus Solanum evolved through 2-oxoglutarate-dependent dioxygenases. *Nat Commun 10*, 5169.

(3) Durr, J., Papareddy, R., Nakajima, K., and Gutierrez-Marcos, J. (2018) Highly efficient heritable targeted deletions of gene clusters and non-coding regulatory regions in Arabidopsis using CRISPR/Cas9. *Sci Rep 8*, 4443.

(4) Yamamoto, T., Hoshikawa, K., Ezura, K., Okazawa, R., Fujita, S., Takaoka, M., Mason, H. S., Ezura, H., and Miura, K. (2018) Improvement of the transient expression system for production of recombinant proteins in plants. *Sci Rep 8*, 4755.

(5) Sarrion-Perdigones, A., Vazquez-Vilar, M., Palací, J., Castelijns, B., Forment, J., Ziarsolo, P., Blanca, J., Granell, A., and Orzaez, D. (2013) GoldenBraid 2.0: a comprehensive DNA assembly framework for plant synthetic biology. *Plant Physiol. 162*, 1618.

(6) Vazquez-Vilar, M., Quijano-Rubio, A., Fernandez-del-Carmen, A., Sarrion-Perdigones, A., Ochoa-Fernandez, R., Ziarsolo, P., Blanca, J., Granell, A., and Orzaez, D. (2017) GB3.0: a platform for plant bio-design that connects functional DNA elements with associated biological data. *Nucleic Acids Res* gkw1326.

(7) Patthy-Lukáts, Á., Kocsis, Á., Szabó, L. F., and Podányi, B. (1999) Configurative correlation and conformational analysis of strictosidine and vincoside derivatives. *J. Nat. Prod. 62*, 1492–1499.

(8) Dührkop, K., Fleischauer, M., Ludwig, M., Aksenov, A. A., Melnik, A. V., Meusel, M., Dorrestein, P. C., Rousu, J., and Böcker, S. (2019) SIRIUS 4: a rapid tool for turning tandem mass spectra into metabolite structure information. *Nat Methods 16*, 299–302.

(9) Pluskal, T., Castillo, S., Villar-Briones, A., and Orešič, M. (2010) MZmine 2: Modular framework for processing, visualizing, and analyzing mass spectrometry-based molecular profile data. *BMC Bioinformatics 11*, 395.

(10) Caputi, L., Franke, J., Farrow, S. C., Chung, K., Payne, R. M. E., Nguyen, T.-D., Dang, T.-T. T., Carqueijeiro, I. S. T., Koudounas, K., Bernonville, T. D. de, Ameyaw, B., Jones, D. M., Vieira, I. J. C., Courdavault, V., and O'Connor, S. E. (2018) Missing enzymes in the biosynthesis of the anticancer drug vinblastine in Madagascar periwinkle. *Science 360*, 1235–1239.

(11) Livak, K. J., and Schmittgen, T. D. (2001) Analysis of relative gene expression data using Real-Time Quantitative PCR and the 2−ΔΔCT method. *Methods 25*, 402–408.
